# Fluorescent macrolide probes – synthesis and use in evaluation of bacterial resistance†

**DOI:** 10.1039/d0cb00118j

**Published:** 2020-11-17

**Authors:** M. Rhia L. Stone, Urszula Łapińska, Stefano Pagliara, Muriel Masi, Joanne T. Blanchfield, Matthew A. Cooper, Mark A. T. Blaskovich

**Affiliations:** Centre for Superbug Solutions, Institute for Molecular Bioscience, The University of Queensland 306 Carmody Road St Lucia 4072 Brisbane Australia m.blaskovich@imb.uq.edu.au; Living Systems Institute, University of Exeter Exeter EX4 4QD UK; Université Paris-Saclay, CEA, CNRS Institute for Integrative Biology of the Cell (I2BC) 911198 Gif-sur-Yvette France; School of Chemistry and Molecular Biosciences, The University of Queensland 68 Cooper Road St Lucia 4072 Brisbane Australia

## Abstract

The emerging crisis of antibiotic resistance requires a multi-pronged approach in order to avert the onset of a post-antibiotic age. Studies of antibiotic uptake and localisation in live cells may inform the design of improved drugs and help develop a better understanding of bacterial resistance and persistence. To facilitate this research, we have synthesised fluorescent derivatives of the macrolide antibiotic erythromycin. These analogues exhibit a similar spectrum of antibiotic activity to the parent drug and are capable of labelling both Gram-positive and -negative bacteria for microscopy. The probes localise intracellularly, with uptake in Gram-negative bacteria dependent on the level of efflux pump activity. A plate-based assay established to quantify bacterial labelling and localisation demonstrated that the probes were taken up by both susceptible and resistant bacteria. Significant intra-strain and -species differences were observed in these preliminary studies. In order to examine uptake in real-time, the probe was used in single-cell microfluidic microscopy, revealing previously unseen heterogeneity of uptake in populations of susceptible bacteria. These studies illustrate the potential of fluorescent macrolide probes to characterise and explore drug uptake and efflux in bacteria.

## Abstract 2

Macrolide fluorescent probes illuminate the interactions between antibiotics and bacteria, providing new insight into mechanisms of resistance.

## Graphical abstract

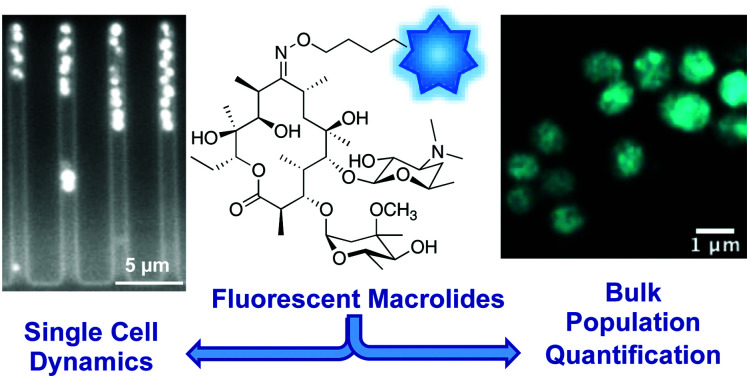

## Introduction

**Fig. 1:**
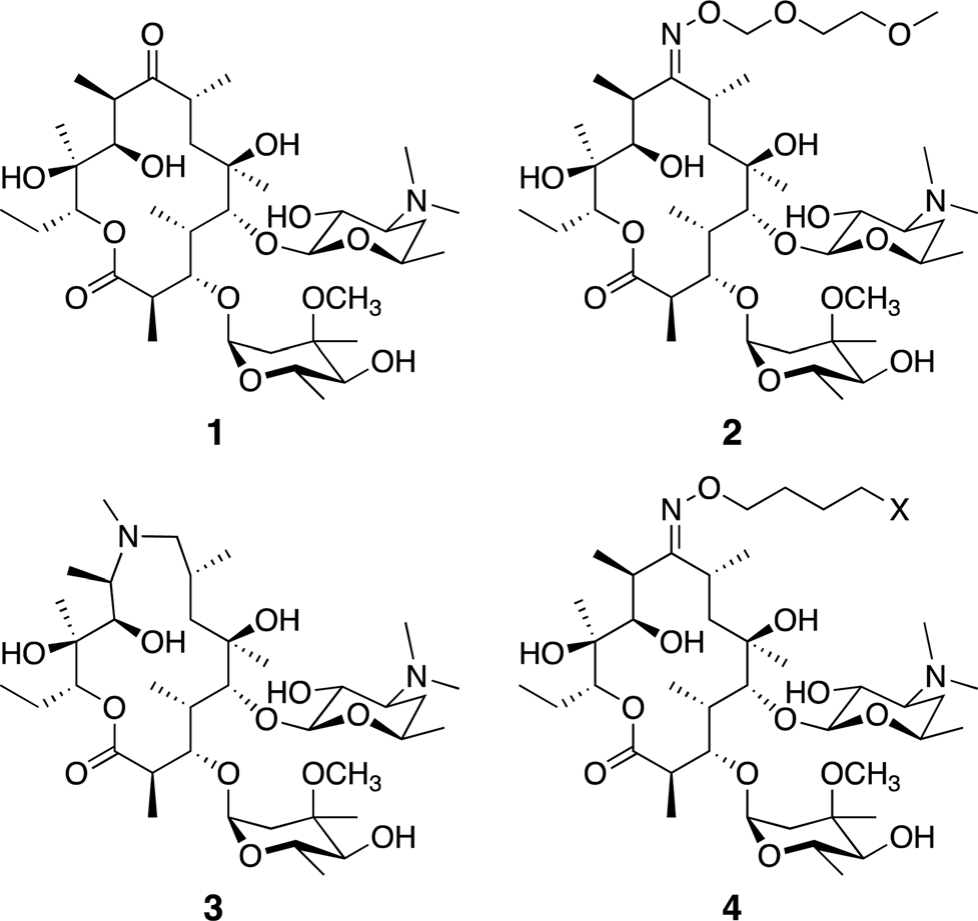
Common macrolide antibiotics erythromycin **1**, roxithromycin **2**, azithromycin **3**, and generic probe structure **4**.

Macrolide antibiotics are a natural product-class of drugs discovered in the mid-twentieth century following isolation from bacteria such as *Saccharopolyspora erythrea*. The archetypal macrolide, erythromycin (**1**, Fig. 1), entered clinical use in 1952. Since then, more than 10 semi-synthetic macrolides (*e.g.* roxithromycin **2** and azithromycin **3**, Fig. 1) have been developed and now see wide-spread clinical use.^1^

Macrolides are generally active against Gram-positive and -negative cocci (*e.g.* Staphylococci, Streptococci, *Neisseria meningitidis*), Gram-positive and some -negative bacilli (*e.g.* Bacilli*, Bordatella*), atypical bacteria (*e.g. Mycobacterium avium* complex, *Helicobacter pylori*), and intracellular pathogens (*e.g. Legionella, Chlamydia*).^2^ One factor increasingly limiting their use is acquired macrolide resistance, which was observed only a few years after their introduction into the clinic.^1^ Resistance to the macrolides falls into one of four mechanistic categories: modification of the macrolide target (the 50 s subunit of the bacterial ribosome); target protection (removal of macrolide from the active site); macrolide efflux (reduces intracellular concentration); or antibiotic modification.^3^ Chief amongst these mechanisms are ribosome modifications effected by the erm (erythromycin ribosome methylation) enzymes, which mono- or di-methylate the ribosome, interfering with macrolide binding; and the mef and mrs efflux pumps, which decrease intracellular concentrations of the antibiotic *via* active export.^1^ Many pathogenic bacteria in the clinic now possess one or more of these resistance mechanisms, with their dissemination between bacteria facilitated by plasmid transmission.^4^

In order to combat macrolide resistance and develop new therapies for resistant bacteria, we need better tools to improve our understanding of drug mode of action and resistance mechanisms, especially in terms of drug accumulation and efflux. One set of tools that have found increasing utility in antibiotic resistance research are fluorescent antibiotics.^5^ Although innately fluorescent or fluorescent analogues of antibiotics were used in the second half of the 20th century to investigate the mode of action of antibiotics,^5^ they have been applied sparingly to help address the modern crisis of resistance. Examples of probes that have been successfully utilised include fluorescent glycopeptides^6^ and β-lactams.^7–9^ However, instead of phenotypic studies, characterisation of resistance has shifted towards – omics studies, where changes at the genetic, epigenetic and transcriptomic levels are analysed.^5^ Where intracellular distribution studies have been conducted, they have largely relied on radioactive analogues, which are costly, difficult to access, and require specialist equipment.^10–19^

Neither of these techniques readily allow for analysis at single-cell resolution, though some single cell sequencing reports are beginning to appear.^20,21^ Despite the success of these approaches, there remains a significant appeal in simple tools that are readily accessible and do not require a large time or resource investment, or which can be utilised in combination with more modern technology, such as high-resolution microscopy and single cell microfluidics.

There have been several fluorescent macrolides reported in the literature, however none of these have seen widespread use. In the 1970s, Pestka and colleagues used a fluorescein-erythromycin conjugate to study interactions with the ribosome,^22,23^ and were able to determine which subunit the probe bound to, along with information about the binding kinetics. These assays were carried out on isolated ribosomes, so it is unclear how the fluorescent macrolide behaved in live bacteria. This is a common flaw with many antibiotic-derived probes – their antibacterial activity is often not reported and the degree of cell penetration unknown.^5^ With the fluorescein substituent being a moderate-sized ligand on its own (*M* = 332 g mol^−1^), it is likely that it would interfere with entry of the conjugate into the cell. Three decades later, Li *et al.* developed boron-dipyrromethene (BODIPY)-erythromycin probes that were also used to study interactions at the ribosome.^24^ Again, antibacterial activity was not measured, so it is unclear how effectively these probes mimic the parent antibiotic. The authors used the fluorescent macrolide to develop a high-throughput screening protocol to identify new antibiotic candidates. More recently, Matijasic *et al.* reported several azithromycin-based fluorescent compounds (with a 7-nitrobenz-2-oxa-1,3-diazol-4-yl (NBD) fluorophore), which were used to evaluate azithromycin distribution in mammalian cells and whole animals.^25^ Alone of the previously reported fluorescent macrolides, those prepared by Matijasic *et al.* were confirmed to retain the antibacterial activity of the parent drug, a critical step in ensuring that the probe accurately represents the parent in studies. However, neither uptake nor localisation in bacterial cells was assessed in their work. To date, no work has been reported utilising fluorescent macrolides for studying antibiotic resistance; indeed there are relatively few reports on the use of any macrolide-derived fluorescent probes.^26^ In order to address this gap, in this paper we report on the synthesis and characterisation of two novel fluorescent derivatives of erythromycin, where a common antibiotic core is linked to two different fluorophores. We assessed their activity against both Gram-positive and Gram-negative bacteria, showing that both probes retained good to moderate levels of antimicrobial activity. In order to assess their utility for the study of antibiotic-bacterial interactions, particularly investigations of resistance mechanisms, we tested their ability to label bacteria using a combination of spectrofluorometry and microscopy, at both single cell and bulk population levels.

For bulk measurements we used spectrophotometry, because this technique is especially useful for quantitative analysis. It is fast, inexpensive, readily available, and compatible with plate-based, high-throughput assays, and allowed us to investigate antimicrobial resistance at the ensemble level. We also employed high-resolution microscopy to gain a more detailed view of interactions between bacteria and the probes at a single cell level. We then bridged both techniques with single-cell microfluidics, where we again gained insights into antimicrobial resistance at the single cell level but were able to simultaneously monitor thousands of individual cells.

## Results and discussion

### Synthesis

**Scheme 1:**
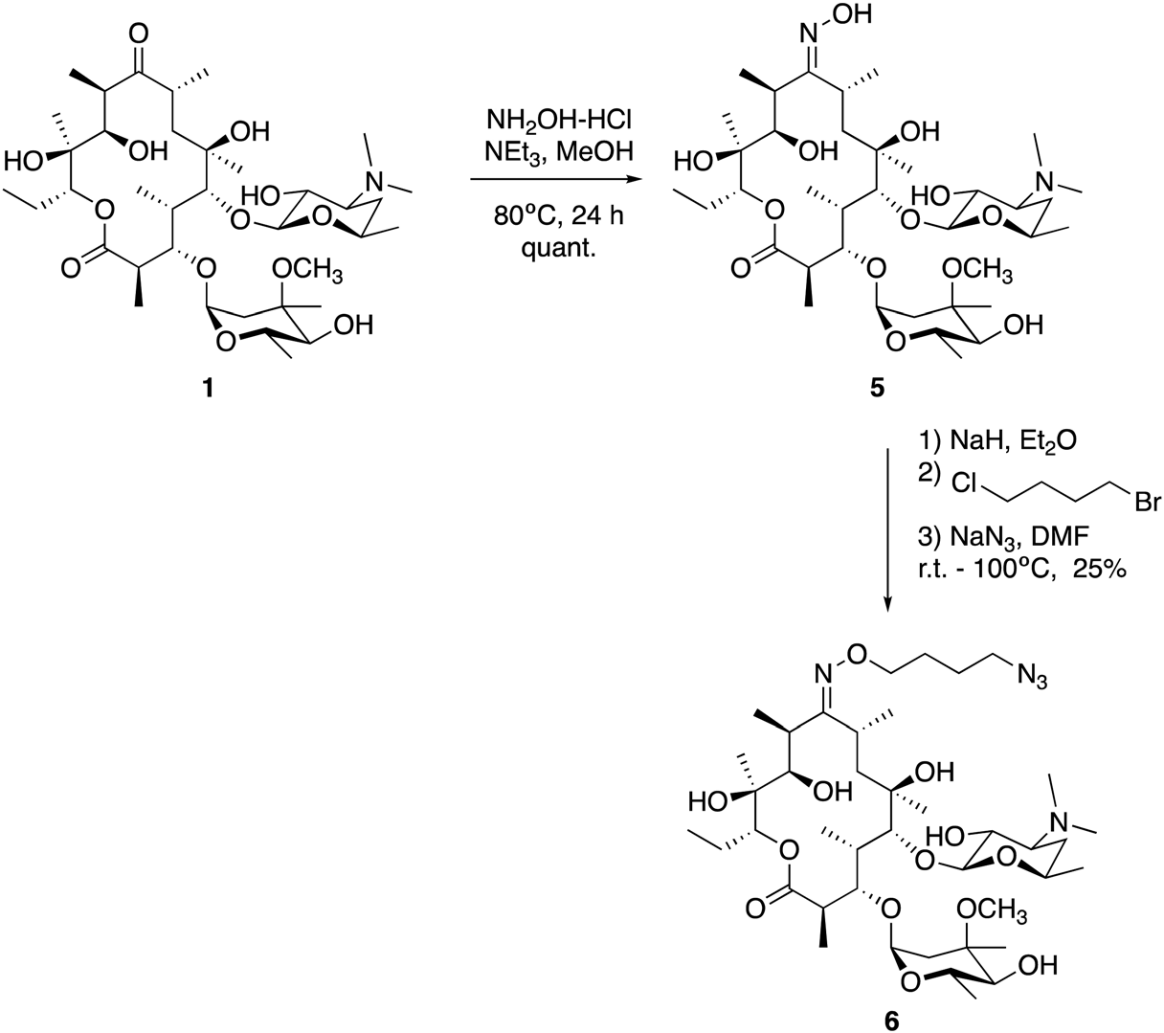
Synthesis of roxithromycin-azide **6** from erythromycin **1**, *via* formation of oxime intermediate **5**, which is then *O*-alkylated with 1-bromo-4-chlorobutane, followed by displacement of the terminal halide with azide.

We utilised the same strategy that we have previously applied to functionalise other antibiotics,^27–29^ whereby we first installed an azide ‘handle’ to which different fluorophores can be readily attached using a copper activated azide–alkyne cycloaddition (CuAAC) reaction. This reaction is highly selective, compatible with unprotected functional groups, and yields a stable, biocompatible triazole linker. A key component of this tactic is to select a site for modification that does not perturb the antibacterial activity. Although several macrolide sites have been modified in the literature, the ketone at the 9-position of erythromycin was selected as the point of attachment due to the simple, high-yielding procedures available (**4**, Fig. 1). This ketone has been observed to not make significant interactions with the ribosomal active site in crystal structure studies.^30^ To this end, erythromycin **1** was treated with hydroxylamine to yield oxime **5** in quantitative yield (Scheme 1).^31^ A linker was then installed by alkylation with 1-bromo-4-chlorobutane. Substitution of the terminal halide with sodium azide, yielding roxi-C_4_–N_3_**6** in 25% yield from the oxime.

**Scheme 2:**
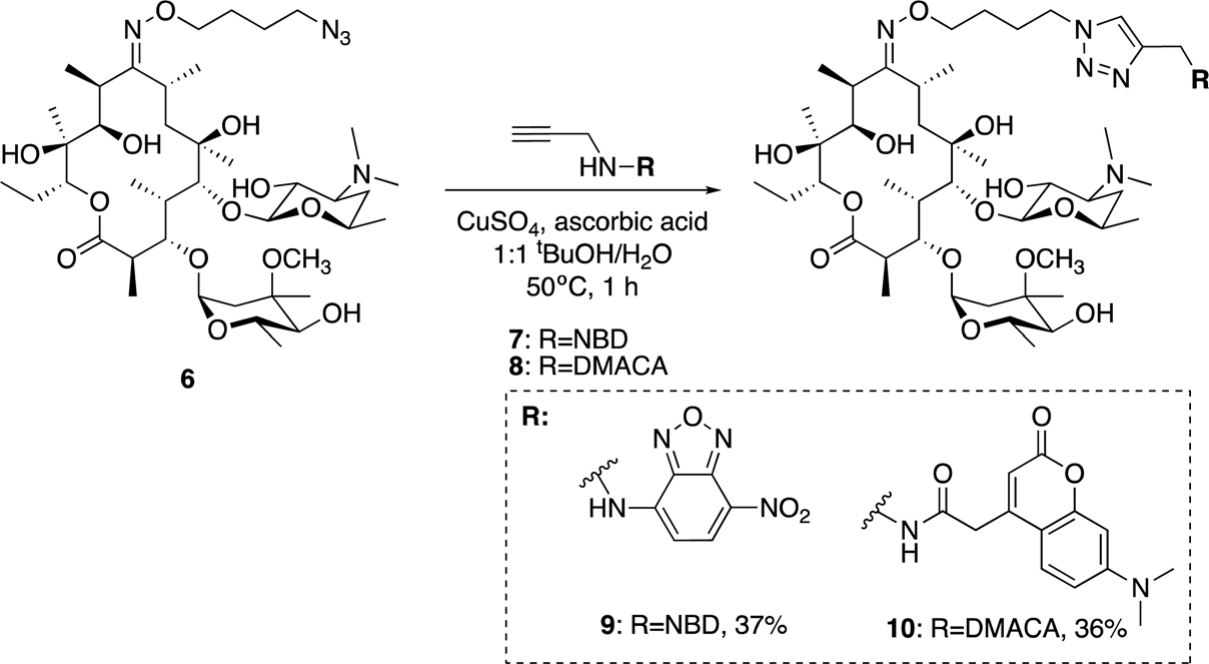
Synthesis of fluorescent macrolides **9** and **10** from azide **6** and fluorophore-alkynes **7** and **8**.

With azide **6** in hand, azide–alkyne click reactions were carried out using alkyne-fluorophores **7** and **8** derived from NBD and 7-dimethylaminocoumarin-4-acetic acid (DMACA) respectively, to form fluorescent macrolides **9** (roxi-C_4_-Tz-NBD) and **10** (roxi-C_4_-Tz-DMACA) in moderate yields (Scheme 2). Elevated temperature was required to facilitate dissolution, and the modest isolated yields (36–37%) were due to product losses during purification on a small scale, rather than incomplete reaction. The NBD and DMACA fluorophores were chosen due to their relatively small size (molecular weight of fluorophore only 165 and 231 g mol^−1^ respectively) compared to other fluorophores such as fluorescein, BODIPY-FL, or rhodamine B (fluorophore MW of 316, 276 and 463 g mol^−1^ respectively). The smaller substituent size reduces the likelihood that attaching it to the antibiotic core will reduce bacterial uptake of the conjugate. Furthermore, conjugation of the NBD fluorophore was found to not impair biological activity for azithromycin,^25^ hence was considered a promising candidate for attachment by click chemistry.

### Antibacterial activity

In order to confirm that the fluorescent probes **9** and **10** retained the antibiotic activity of the parent drug, minimum inhibitory concentrations (MICs) were measured against a panel of susceptible bacteria (Table 1). Here, roxi-C_4_–N_3_**6** displayed good to excellent antibacterial activity against all Gram-positive cocci and bacilli tested, with MICs generally at most 8-fold less potent compared to the parent drugs erythromycin **1** and roxithromycin **2**. Similarly, NBD probe **9** exhibited good to moderate antibacterial activity, with very similar activity as the azide **6** against Staphylococci and Streptococci (*e.g.* 4- to 8-fold less active than the parent roxithromycin). The DMACA probe **10** was also active at inhibiting bacterial growth, though was less potent than the other derivatives tested, with approximately 4- to 16-fold increased MIC compared to the NBD probe. However, both probes showed similar relative changes in potency across the different strains tested as the parent antibiotic. For example, consistent with the lack of activity of erythromycin and roxithromycin (MIC ≥ 64 μg mL^−1^), none of the macrolide probes showed substantial inhibitory activity against the one Gram-negative species tested, *Escherichia coli* (*E. coli*).

**Table 1:** Minimum inhibitory concentrations (MICs, in μg mL^−1^) of erythromycin (**1**), roxithromycin (**2**), roxi-C_4_–N_3_ (**6**), roxi-C4-Tz-NBD (**9**), and roxi-C4-Tz-DMACA (**10**) against a panel of American Type Culture Collection (ATTC) susceptible bacteria, measured by broth microdilution in cation-adjusted Mueller Hinton broth (CAMHB), *n* ≥ 4

Species	Strain	Ery 1	Roxi 2	Roxi-N_3_ 6	Roxi-NBD 9	Roxi-DMACA 10
*S. aureus*	ATCC25923	0.25–0.5	0.25–2	1–4	1–4	8–16
*S. epidermidis*	ATCC12228	0.25–1	0.5–1	0.031–0.063	0.5–1	16–64
*S. pneumoniae*	ATCC33400	0.25–0.5	0.5–2	1–2	1–2	8–32
*S. pyogenes*	ATCC12344	≤0.016	≤0.016	≤0.016	0.031–0.063	0.13
*E. faecium*	ATCC35667	0.25–2	1–8	2–8	16	64
*B. subtilis*	ATCC6051	0.13–0.25	0.25–2	0.5–1	1–2	8 -16
*E. coli*	ATCC25922	64	≥64	≥64	≥64	≥64
	AG102^a^	≥64	≥64	≥64	≥64	≥64

a *mar1*, upregulated Acr-AB efflux pump.

A panel of resistant Streptococci with either defined (*erm(B)*+, *mef(e)*+) or undefined mechanisms of resistance were then tested (Table 2). The *Streptococcus pneumoniae* (*S. pneumoniae*) strains were found to be highly resistant to all the macrolides tested, with MICs of ≥64 μg mL^−1^ recorded. This was also the case for *S. pyogenes* ATCC BAA-1412, however the MICs against ATCC BAA-1414 were only mildly elevated (still above breakpoint for roxithromycin **2**).^32^ Overall, the azide intermediate and fluorescent probes had similar antibiotic activity profiles as compared to the parent drugs, with greatly increased MICs against the macrolide-resistant bacteria. The MIC values were used to inform appropriate concentrations for subsequent assays.

**Table 2:** Minimum inhibitory concentrations (MICs, in μg mL^−1^) of erythromycin (**1**), roxithromycin (**2**), roxi-C_4_–N_3_ (**6**), roxi-C_4_-Tz-NBD (**9**), and roxi-C_4_-Tz-DMACA (**10**) against a panel of susceptible (for comparison) and resistant Streptococci, measured by broth microdilution in brain-heart infusion (BHI), *n* ≥ 3

Species	Strain	Ery 1	Roxi 2	Roxi-N_3_ 6	Roxi-NBD 9	Roxi-DMACA 10
*S. pneumoniae*	ATCC 33400	0.25–0.5	0.25–2	1–4	1–4	8–16
	ATCC 700677^a^	≥64	16–≥64	16–≥64	4–≥64	8–≥64
	ATCC 700676^b^^c^	≥64	≥64	≥64	≥64	≥64

*S. pyogenes*	ATCC 12344	≤0.016	≤0.016	≤0.016	0.031–0.063	0.13
	ATCC BAA-1412	≥64	≥64	≥64	≥64	≥64
	ATCC BAA-1414	0.031–0.13	1–2	0.063–1	≤0.016–0.063	1

a *erm(B)*+.

b *mef(e)*+.

c 5% CO_2_.

### Intracellular labelling and localisation

**Fig. 2:**
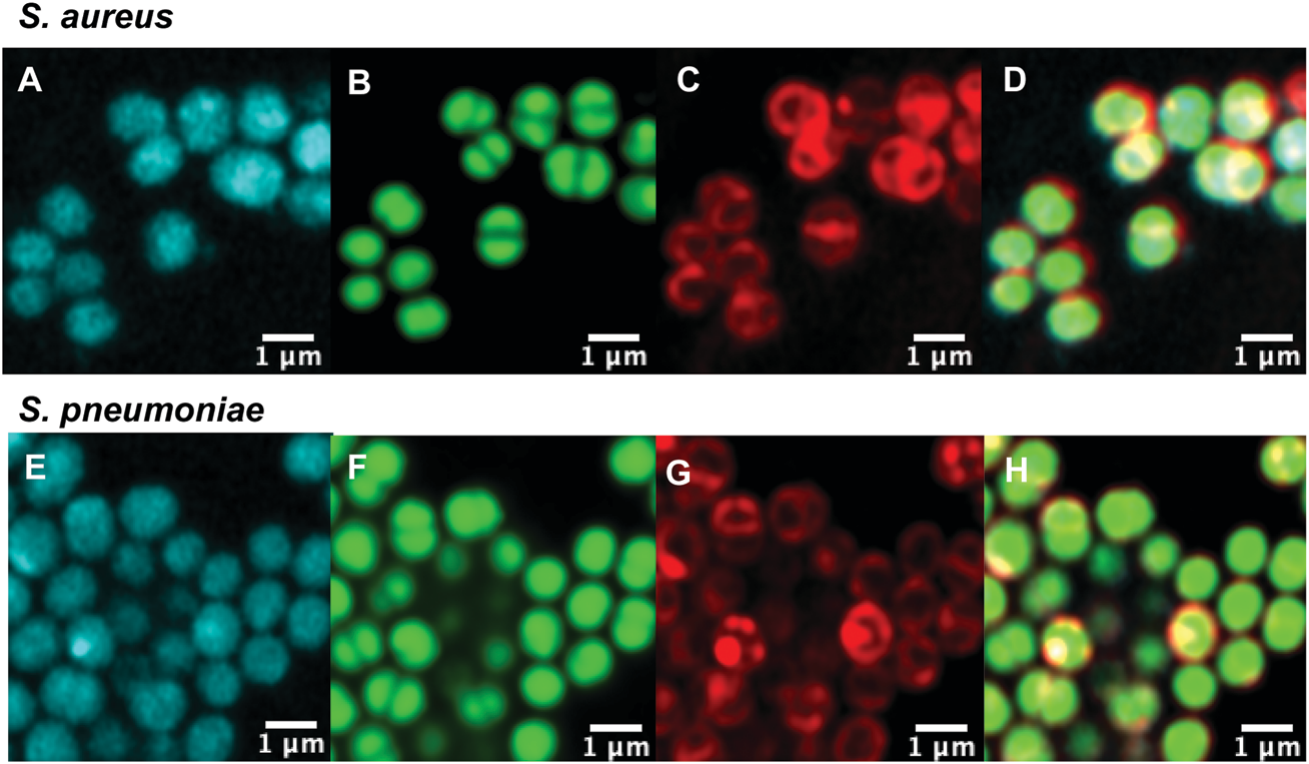
Confocal microscopic images of (A–D) live susceptible *S. aureus* (ATCC 25923) and (E–H) susceptible *S. pneumoniae* (ATCC 33400) labelled with (A and E) roxi-C_4_-Tz-DMACA **10** (10 μM); (B and F) Syto-9 (green nucleic acid dye, 5 μM); (C and G) FM4-64FX (red membrane dye, 5 μg mL^−1^); and (D and H) overlaid.

With antibiotic activity established, live-cell confocal microscopy was performed in *S. aureus* and susceptible and resistant Streptococci to examine localisation and labelling and gain insight about the macrolide resistance mechanisms in resistant Streptococci strains. Bacteria were incubated with fluorescent probes **9** and **10**, along with the red membrane dye FM4-64FX and a complementary nucleic acid dye (Syto-9, green; or Hoechst-33342, blue). Despite its reduced antibacterial potency, Roxi-C_4_-Tz-DMACA **10** was found to provide superior labelling for microscopy and was able to be used at concentrations as low as 1 nM in some species. The reason for this difference from NBD probe **9** is unclear but given the similarity in MIC (Tables 1 and 2) and uptake results from the quantitative assays (see Fig. 5, 7 and Table 3 below), the results obtained from **10** should be representative of both probes, and the parent antibiotic. Susceptible strains of *Staphylococcus aureus* (*S. aureus*) and *S. pneumoniae* were first examined (Fig. 2), with *S. pyogenes* (Fig. S1, ESI†) showing similar results, where it was found that **10** localised intracellularly. This is in contrast to the fluorophore-alkynes, which did not significantly accumulate in the bacteria (Fig. S2, ESI†).

**Table 3:** Percentage of fluorescence of probes **9** and **10** in the IF from (IF + pellet)

Species	ATCC	S/R^a^	Average IF/(IF + pellet) (%)	
			DMACA probe **10**	NBD probe **9**
*S. aureus*	25923	S	89	87
*S. pyogenes*	12344	S	83	64
	BAA-1412	R	68	38
	BAA-1414	R	83	78
*S. pneumoniae*	33400	S	87	90
	700677^b^	*R*	*94*	*99*
	700676^c^	R	96	96

a S: susceptible, R: resistant.

b *erm(B)*+.

c *mef(e)*+; *italics*: highly variable due to poor absorption.

**Fig. 3:**
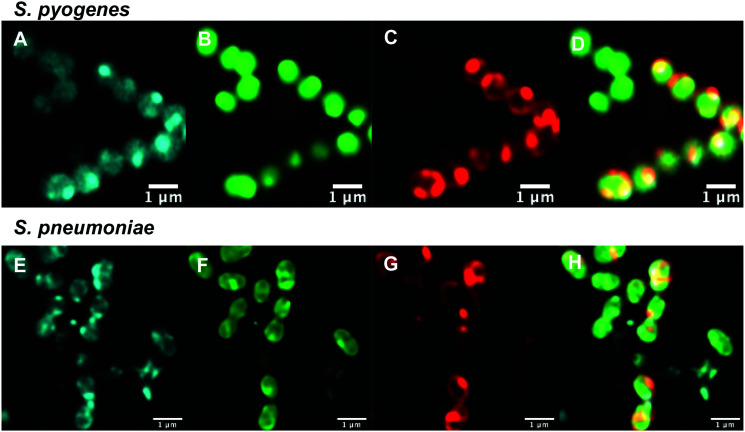
Confocal microscopic images of (A–D) live resistant *S. pyogenes* (ATCC BAA-1412) and (E and F) resistant *S. pneumoniae* (ATCC 700676) labelled with (A and E) roxi-C_4_-Tz-DMACA **10** (10 μM); (B and F) Syto-9 (green nucleic acid dye, 5 μM); (C and G) FM4-64FX (red membrane dye, 5 μg mL^−1^); and (D and H) overlaid.

After confirming that the macrolide probe **10** successfully reached the bacterial cytoplasm in susceptible strains, we analysed its localization in resistant *S. pyogenes* and *S. pneumoniae*. Surprisingly, we found that **10** was again found to localise in the cytoplasm (Fig. 3 and Fig. S5, ESI†), including in *mef(e)*+ *S. pneumoniae*. The latter strain, equipped with upregulated mef efflux pumps, appeared to show patterns of localised intensity when compared to the corresponding susceptible strain, but still had substantial internal labelling. In order to assess whether this difference in labelling was due to increased efflux in the resistant strain, samples were incubated with the protonophore carbonyl cyanide *m*-chlorophenylhydrazine (CCCP), which acts by disabling the proton motive force driving efflux pumps.^33^ In this case we found no change in the localisation or intensity of labelling compared to the susceptible strain ATCC 33400.

### Dynamics of intracellular accumulation

**Fig. 4:**
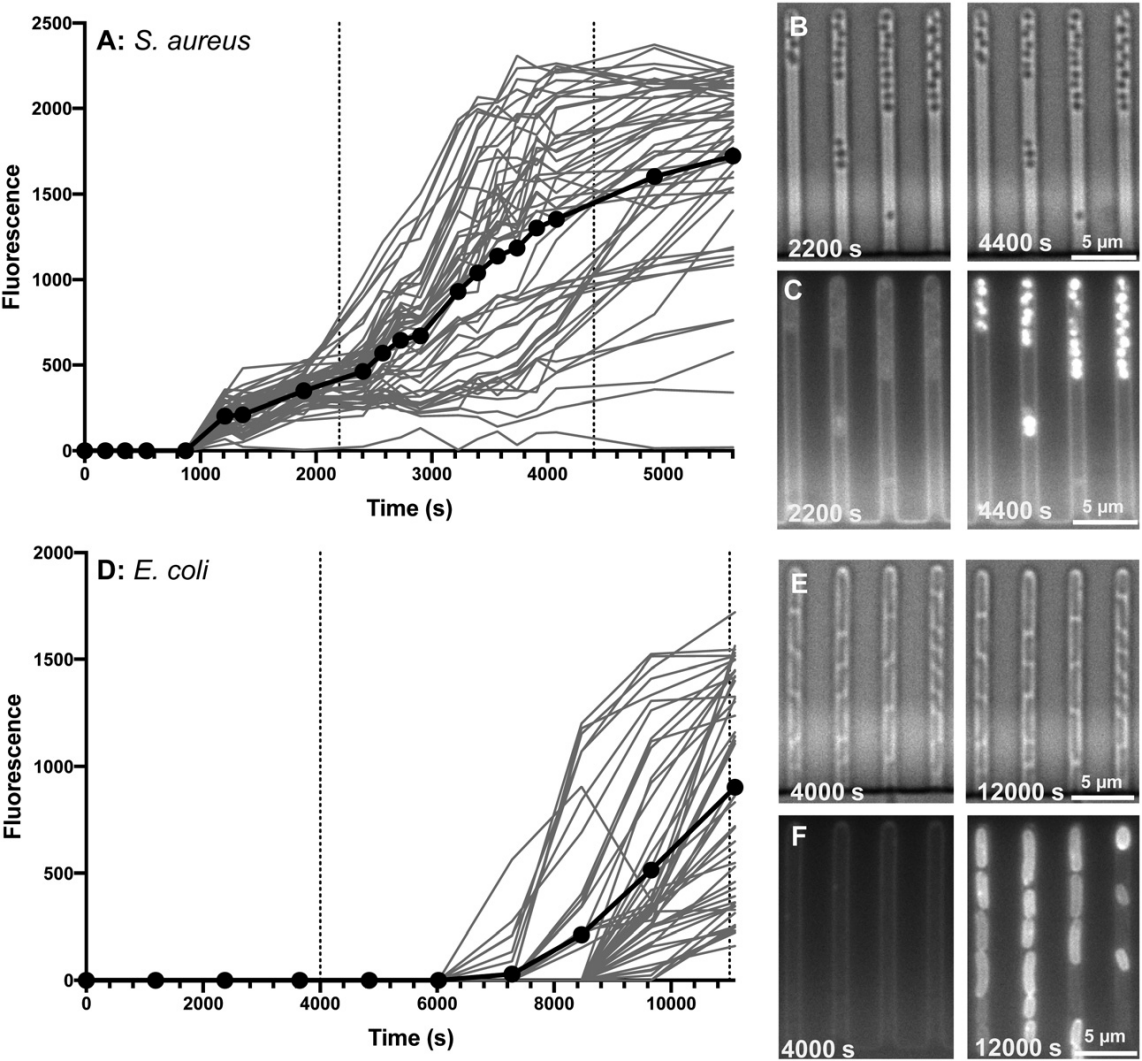
Accumulation of roxi-C_4_-Tz-NBD **9** in susceptible *S. aureus* (MSSA476) (A–C) and *E. coli* (BW25113) (D–F) cells as monitored by single-cell microfluidics over time. Fluorescence of individual cells is tracked in (A and D), with the average shown in bold with symbols. Times of snapshots (B, C and E, F) are indicated with dashed vertical lines. Brightfield (B and E) and the corresponding fluorescent (C and F) images are shown at early (2200 and 4000 s) and late (4400 and 12 000 s) timepoints for both species.

Although confocal microscopy allows for observation of the precise intracellular localisation of the macrolide probes, it is not suitable for quantifying the dynamics of antibiotic accumulation in individual bacteria. Therefore, we complemented the measurements above with studies carried out using our previously established single-cell microfluidics-microscopy assay.^34–37^ This technique enables single-cell studies to address the impact of phenotypic heterogeneity^38,39^ on intracellular drug accumulation and efficacy.^40,41^ As a proof of concept, we examined the real-time uptake of roxi-C_4_-Tz-NBD **9** into *E. coli* (BW25113, a K12 strain) and susceptible *S. aureus* (MSSA476). Here it was found that the probe was rapidly taken up by *S. aureus* cells, with uptake initiated near-simultaneously across all measured cells, although there was significant variation in the speed and extent of uptake (Fig. 4A). In contrast, there was a long lag period before any *E. coli* became fluorescent (Fig. 4B). Even when the *E. coli* did take on the probe, both initiation and rate of uptake showed a substantial variability in response. The spread of fluorescence behaviour indicates that in both species examined, there is inherent population variability in response to the roxi-NBD probe **9**. For *E. coli*, there were also cells which did not accumulate any probe, despite being surrounded by bacteria with substantial uptake (see Fig. 4E and F, *e.g.* right hand panels, third channel, 2nd bacterium from the bottom is visible in the brightfield microscopy image, but is not fluorescent despite cells on either side with high levels of fluorescence). The intracellular variation is striking. This suggests heterogeneity in the macrolide response, potentially due to differences in levels of native efflux pumps or in the abundance of ribosomes that are the intracellular targets of macrolides, or a quiescent subpopulation. These new probes and time-dependent data open the way for deepening our understanding of subsets of bacteria, including persister and viable but non culturable cells,^40,42^ that can transiently survive antibiotic exposure and contribute to recalcitrant infections^43^ and the development of antibiotic resistance.^44^

### Dynamics of efflux

In order to gain further insight on the molecular mechanisms underlying reduced intracellular accumulation of our macrolide probes, we used ensemble measurements to quantify efflux, one of the major forms of resistance to macrolides. We hoped that the macrolide probes could be used as tools to identify efflux upregulation in resistant bacteria, study this mode of resistance, and help guide the development of strategies to overcome efflux.

**Fig. 5:**
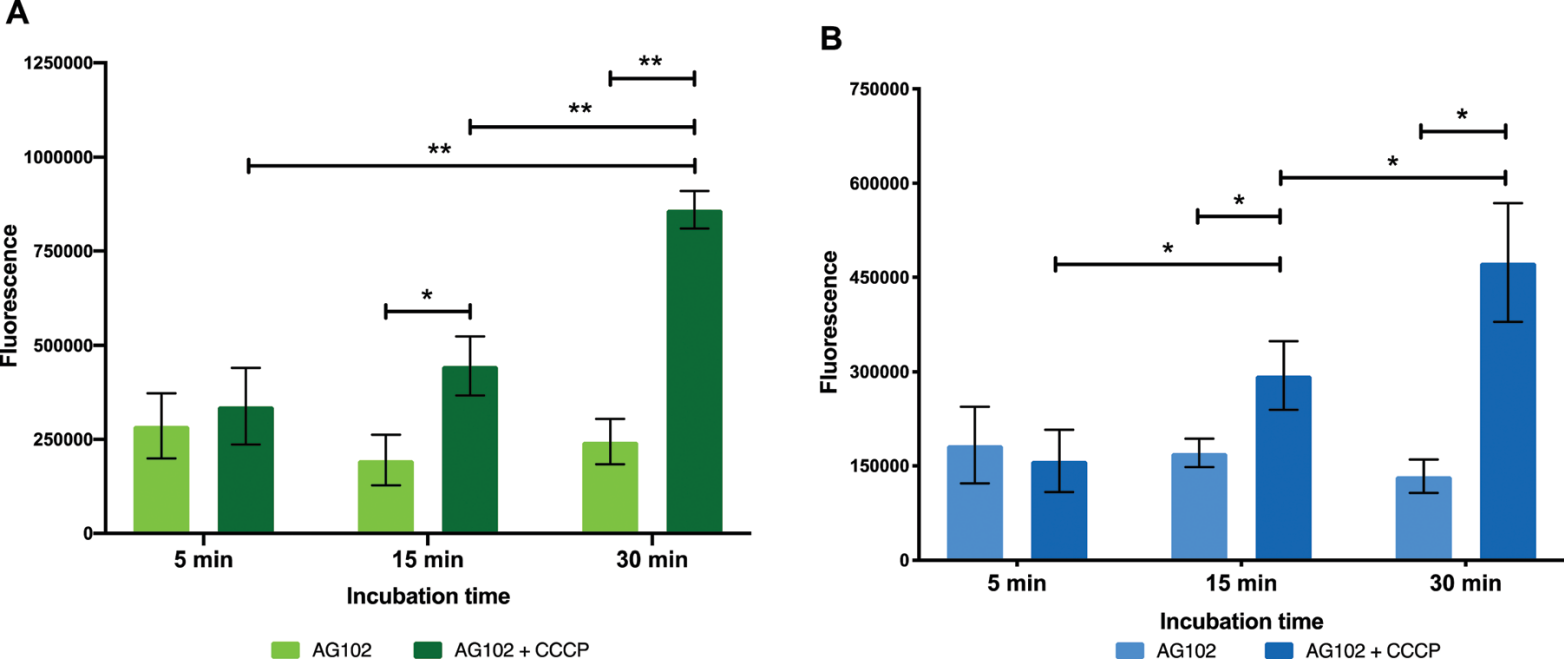
Intracellular accumulation of macrolide probes with NBD **9** (A) and DMACA **10** (B) fluorophores (50 μM) in AG102 *E. coli* in the absence and presence of efflux inhibitor CCCP (10 μM), *n* = 3, * = *p* < 0.05, ** = *p* < 0.01.

To start the examination of bacterial efflux of fluorescent macrolides **9** and **10**, the model organism *E. coli* AG102 was used, in which efflux pump AcrAB is overproduced (*mar1* mutant). In order to assess whether the roxi-probes were subject to efflux in this model, intracellular accumulation was measured by spectrophotometry over time in the presence and absence of CCCP (Fig. 5). Mid-log phase bacteria were incubated with the macrolide probes (with or without CCCP), with aliquots withdrawn at each time point. Labelled bacteria were then collected and lysed overnight using glycine-HCl. The lysed bacteria were centrifuged, and the fluorescence of the decantate measured using a plate reader. As expected, neither roxi-C_4_-Tz-NBD **9** or roxi-C_4_-Tz-DMACA **10** showed significant internalisation into AG102 in the absence of CCCP. In contrast, both probes showed a time-dependent accumulation when efflux was abolished. This finding indicates both that the fluorescent macrolides are indeed efflux pump substrates (potentially the AcrAB pump in this strain of *E. coli*) and their internal accumulation can be used as a marker for the presence of efflux.

**Fig. 6:**
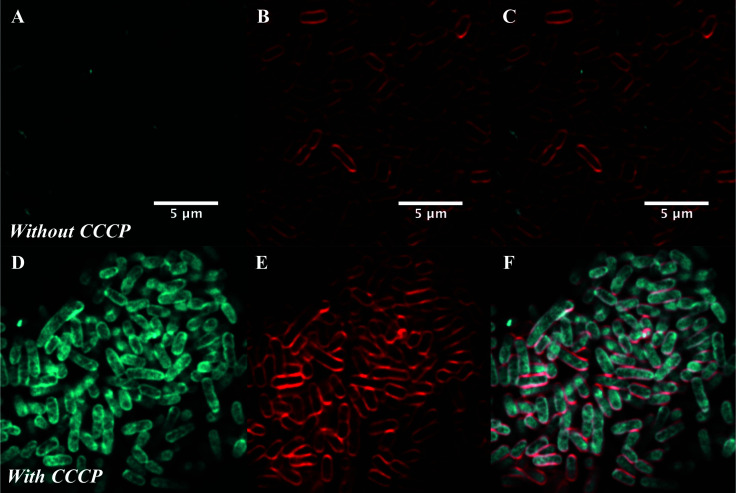
Airyscan confocal microscopy of AG102 *E. coli* without (A–C) or with (D–F) treatment with 10 μM CCCP: (A and D) DMACA probe **10** (5 μM), (B and E) FM4-64FX (red membrane stain), and (C and F) overlaid.

To confirm this, live cell microscopy was carried out with AG102 incubated with roxi-C_4_-Tz-DMACA **9** with or without CCCP (Fig. 6). Here, the same pattern of labelling was observed, with the efflux-active cells not showing significant uptake of the probe, but CCCP-treated efflux-negative cells having considerable internal probe fluorescence. Gratifyingly, this lack of significant labelling in efflux-active *E. coli* qualitatively corroborates the single-cell data concerning the dynamics of macrolide accumulation presented in Fig. 4. Although eventual uptake was seen in microfluidic analysis, this was not until after 80 min, whereas the microscopy samples were only incubated with probes for 30 min.

### Quantification of intracellular labelling

**Fig. 7:**
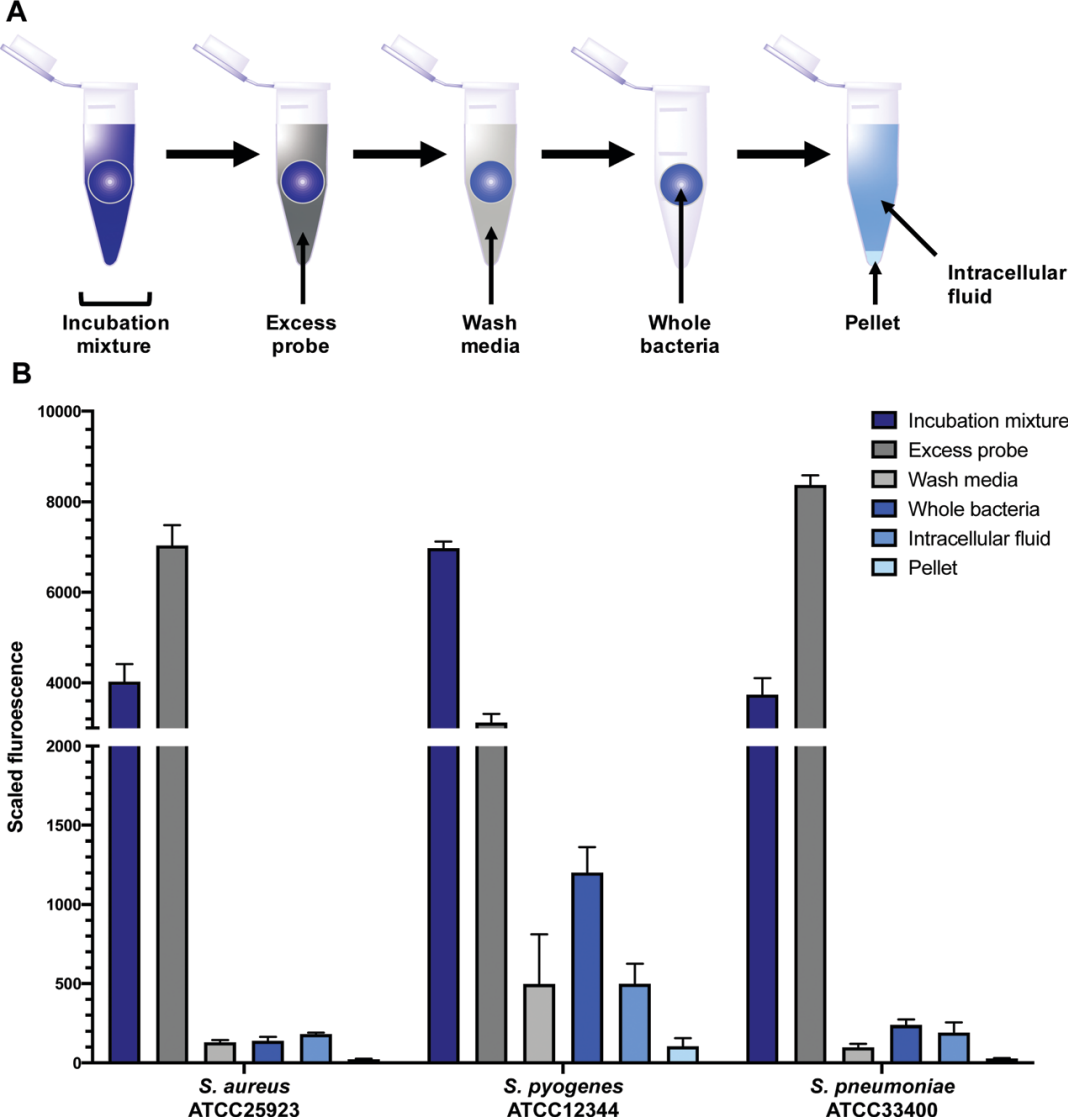
Scaled fluorescence of roxi-C_4_-Tz-DMACA **10** in aliquots (incubation mixture, excess probe, wash media, whole bacteria, intracellular fluid, pellet) of different bacteria: susceptible *S. aureus* and susceptible *S. pyogenes* and *S. pneumoniae*.

In order to quantitatively evaluate intracellular labelling in a high-throughput bulk-population measurement, a plate- based assay was developed using *S. aureus*, *S. pneumoniae*, and *S. pyogenes*, building on existing work in the literature.^45^ Bacteria were grown to mid-log phase, then treated with probes **9** or **10**, along with controls of alkynes **7** and **8**, and roxithromycin **2**. Aliquots were taken of this incubation mixture, which was then centrifuged. Another aliquot was taken of the supernatant, which contained excess probe not taken up by the bacteria (Fig. 7). The bacteria were then washed, and portions of both the wash decantate (containing loosely bound probe that could be washed off, or probe that may have been rapidly effluxed after uptake) and the washed bacteria were taken for sampling (see ESI† for more detail). Finally, the remaining washed bacteria were lysed using lysozyme, detergent (bacterial protein extraction reagent, B-PER) and freeze-thawing, then centrifuged to separate the intracellular fluid (IF) (*e.g.* with any free probe inside the bacteria) from the remaining pellet (with probe bound to cell membrane or intracellular components).

All fractions were then assessed for fluorescence. In order to transform the raw data into meaningful values, several normalisations were conducted (see ESI†). At the 1 μM concentration used in the assay (∼1 μg mL^−1^), roxi-C_4_-Tz-DMACA **10** was found to mostly elute in the excess probe aliquot, indicating this is beyond the saturation limit of the bacteria (Fig. 6). Given that the concentration to which the bacteria were exposed is around (susceptible) or below (resistant) the MIC, it indicates that uptake is a significant limiting factor in antibacterial activity. The next step, washing, did not lead to significant loss of fluorescence, showing that once absorbed, the probe is not readily removed. As similarly low fluorescence levels were seen in the wash of efflux active strains, it can be inferred that substantial macrolide efflux does not occur over this timeframe. From the whole washed bacteria (medium blue bars in Fig. 6), lysis revealed that the probe fluorescence was primarily localised in the intracellular fluid, rather than bound to cellular debris in the pellet fraction. This is consistent with the probes targeting the ribosomes, which are normally located in the intracellular fluid. The labelling and localisation of roxi-C_4_-Tz-NBD **9** proved to be very similar to its DMACA counterpart **10** (Fig. S4, ESI†). As before, most of the fluorescence was observed in the excess aliquot, though this was less prominent in *S. pyogenes*, possibly due to increased uptake of the NBD probe. Fluorophore alkynes **7** and **8** were also tested as controls, but they washed off the bacteria with practically no uptake from the full to whole aliquots, confirming that the roxithromycin fluorescent probes are acting in a target specific fashion (Fig. S4, ESI†).

In order to quantify the uptake of the probes, we calculated the percentage of the (reconstructed) full signal that ended up in the intracellular fluid (for details see ESI†). The percentage of probe taken up by the bacteria was found to be highly variable between different species, ranging from 2% of the total added in susceptible *S. aureus* to as much as 50% in *S. pyogenes* (Table S6, ESI†). In general, the values obtained for the two fluorescent macrolide probes were similar, and in contrast the fluorophore-alkynes showed very little uptake in any of the bacteria tested.

As mentioned previously, the macrolide probes localised in the cytosol. Of the combined cytosol and pellet fluorescence obtained after lysis, the IF portion contributed greater than 75% in most cases (Table 3). The percentages for roxi-C_4_-Tz-DMACA **10** and roxi-C_4_-Tz-NBD **9** were generally very comparable in *S. aureus* and *S. pneumoniae*, but in *S. pyogenes* the NBD probe **9** consistently showed lower levels of accumulation, indicating a potential species-selective bias between the NBD *vs.* DMACA probes.

Comparing the macrolide sensitive and resistant Streptococci, contrasting trends were again observed between *S. pyogenes* and *S. pneumoniae*. ATCC BAA-1412 resistant *S. pyogenes* showed a reduced fluorescence in the IF aliquot, potentially indicating less of an attraction for this subcellular localisation. In contrast, the *mef(e)*+ *S. pneumoniae* ATCC 700676 showed a somewhat increased preference for localisation of the probe in the IF compared to the susceptible strain (Table 3) and a similar overall uptake (IF and pellet, Fig. S2, ESI†), an unexpected result given that greater efflux should lead to reduced intracellular levels, if the efflux is responsible for the macrolide resistance. However, the result is consistent with the uptake seen in confocal microscopy (Fig. 3*vs.*Fig. 2), suggesting that the macrolide probe may not actually be a substrate for the *S. pneumoniae* efflux pumps, with the higher MIC due to an alternative resistance mechanism.

These results, albeit preliminary, illustrate the power of this assay in generating data on the localisation of antibiotics, especially when applied to investigations of resistance. Combining qualitative high-resolution fluorescence microscopy with quantitative single-cell and bulk-population methods and mechanistic-specific fluorescent probes can provide a rich understanding of the chemical biology of the interactions between bacteria and antibiotics.

## Conclusion

Two novel fluorescent derivatives of roxithromycin have been synthesised from erythromycin, *via* site-selective modification to add an azide substituent which was then utilised for azide–alkyne ‘click’ chemistry to link two different colour fluorophores with an alkyne substituent. The resultant probes retained the same pattern of antibiotic activity as the parent drugs, and labelled both Gram-positive and -negative bacteria when visualised by confocal microscopy. Fluorescence was observed to be localised inside the bacteria, with greatly increased uptake observed in efflux-inhibited Gram-negative bacteria. However, reduced uptake was not seen in efflux upregulated Gram-positive bacteria. In order to examine the dynamics of the accumulation of these probes, single-cell microfluidics was used, revealing significant heterogeneity within populations that may relate to persistence and/or resistance. Lastly, uptake was quantified on the bulk-scale by a plate-based assay. The probes were taken up by the bacteria and mostly localised in the intracellular fluid. Significant differences were seen between different species and strains, with unexpected trends in some strains with upregulated efflux, indicating scope for future work. This preliminary work illustrates the potential utility of the macrolide fluorescent probes in illuminating the interactions between antibiotics and bacteria, providing new insight into mechanisms of resistance. Fluorescent probes enable the researcher to visualise target interactions in a practical manner, and can be applied to experiments that range from studies of isolated targets through to investigations of mixed populations. Future implications include quantitatively distinguishing modes of macrolide resistance and uncovering heterogeneity in antibiotic response amongst bacterial populations.

## Conflicts of interest

There are no conflicts to declare.

## Supplementary Material

CB-001-D0CB00118J-s001
