# Supplementary material for: Fluorescent macrolide probes – synthesis and use in evaluation of bacterial resistance
Source: RSC Chem Biol. 2020 Nov 17;1(5):395–404. doi: 10.1039/d0cb00118j (PMC8341779; doi:10.1039/d0cb00118j)
Supplement: CB-001-D0CB00118J-s001 [file CB-001-D0CB00118J-s001.pdf]

Electronic Supplementary Material (ESI) for RSC Chemical Biology.
This journal is © The Royal Society of Chemistry 2020

Supplementary Information

Experimental

Materials and Methods

Reagents and non-anhydrous solvents were used as received from the supplier. Dry Et_2O was purified by an Innovative Technology Pure Solv, all other anhydrous solvents were used as received. Anhydrous reactions were performed under nitrogen atmosphere. Reaction progress was monitored by analytical liquid chromatography mass spectrometry (LCMS), performed on an Agilent 1200 series LCMS using 0.05% formic acid (FA) in water as solvent A and 0.05% FA in acetonitrile (ACN) as solvent B. Standard method schedules are as follows: C4T07 (Eclipse Phenyl, Agilent, 0.3 min 5% B, 3.2 min 100%, 5.2 min 100%, 5.5 min 5%, 7.00 min 5%), C4T11 (Eclipse Phenyl, Agilent, 0.5 min 5% B, 7.5 min 100%, 9.0 min 100%, 9.5 min 5%, 11 min 5%), C4T13 (Eclipse Phenyl, Agilent, 0 min 5% B, 9 min 100%, 10 min 100%, 10.2 min 5%, 13 min 5%). Purification was done by normal phase flash chromatography on silica gel (230 – 400 mesh), reverse phase medium-pressure liquid chromatography (MPLC, Reveleris GRACE, Reveleris C-18 Reversed-Phase 12 g or 4 g) and/or high-pressure liquid chromatography (HPLC) (Gilson HPLC 2020, Atlantis Prep T3 OBD (5 or 10 μm, 19 x 100 mm or 10 x 250 mm), Eclipse XBD-Phenyl PrepHT 5 μm 21.2 x 100 mm, or Xterra Prep RP18 OBD 5 μm 19 x 100 mm). Detection was carried out at 210 or 254 nm, or by MS analysis. High-resolution mass spectrometry (HRMS) was carried out on an Agilent 1290 Infinity II or 6545 Q-TOF LCMS. or a Bruker Micro TOF mass spectrometer using (+)- electron spray impact (ESI) calibrated to NH_4OAc. NMR data were collected and calibrated in d_6-DMSO or CD_3OD at 298 K on a Bruker Avance 600 MHz spectrometer.

Microbiology work was carried out in a biocontainment hood. Bacteria isolates were obtained from the American Type Culture Collection (ATCC; Manassas, VA, USA). Tryptic soy agar (TSA) (Becton-Dickson), cation-adjusted Mueller Hinton broth (CAMHB, Becton Dickson), and brain-heart infusion (BHI, Thermo-Fisher) were prepared according to manufacturer instructions then autoclaved. Lysogeny broth (LB) media (except for that used in microfluidics) and agar and phosphate-buffered saline (PBS) were obtained pre-sterilised from the IMB Central Sterilising Facility. Pipette tips were autoclaved before use, and sterile consumables used. Standard polystyrene 96-well plates were used for minimum inhibitory concentration (MIC) assays. Sterile 96-well black flat bottomed polystyrene plates were used for fluorescence

spectrophotometry. Sterile 2 mL capacity 96-well plates were used for the quantification assay. Optical density was measured at 600 nm, and spectrophotometry was carried out on a Tecan Infinite M1000 Pro. High performance glass cover slips by Schott/Zeiss (18 x 18 mm) and superfrost glass microscope slides by Menzel (26 x 76 mm), and Vectashield or Cygel mounting medium were used. Microscopy was carried out on a Leica STED 3X Super Resolution Microscope with White Light Laser excitation or Zeiss Axiovert 200 with Fast Airyscan Detectors.

Synthetic Methodology

Erythromycin-9-oxime ***5***

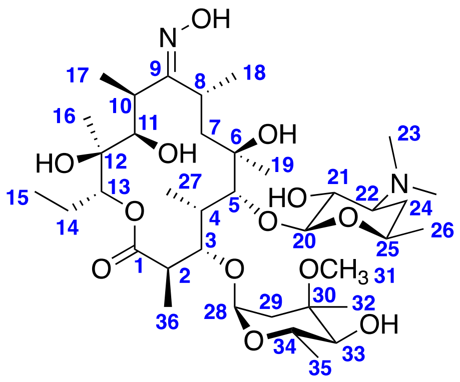

Following methodology developed by Gasc *et al.*[1], erythromycin **1** (5.024 g, 6.849 mmol) was dissolved in 10 mL methanol, then NEt_3 (2.400 mL, 17.23 mmol) was added, followed by $NH_2OH{\cdot}HCl$ (2.375 g, 34.18 mmol). The reaction was heated to 80 °C for 24 h, then cooled to 4 °C for 1 h. The suspension was filtered, and the white solid collected. Product was dried by evaporation under reduced pressure, then co-evaporated with ethanol to give erythromycin-9-oxime **5** (5.206 g, quantitative). LCMS: R_t = 4.62 min, C4T11, $[M+H]^+$ = 749.4; HRMS (ESI, +) $C_{37}H_{69}N_2O_{13}$ $[M+H^+]^+$ calculated 749.4794, observed 845.5385, error 3.34 ppm; IR (thin film, cm^{-1}) λ 3550-3000 (broad, O-H), 2950-2900 (broad, C-H), 1701 (C=O), 1654 (C=N), 1231 (C-N), 1063 (C-O), 1027 (C-O); ^{1}H NMR (600 MHz, d_6-DMSO) δ 5.89 (s, 1H), 5.12 (dd, $^3J_{13,14a}$ = 11.1 Hz, $^3J_{13,14b}$ = 2.3 Hz, 1H, H-13), 4.74 (d, $^3J_{28,4}$ = 5.0 Hz, 1H, H-28), 4.45 (d, $^3J_{20,21}$ = 7.2 Hz, 1H, H-20), 4.36 (d, $^3J_{33\text{-OH},33}$ = 7.7 Hz, 1H, H-33-OH), 4.09 (s, 1H, H-6/12 OH), 4.04 (s, 1H, H-6/12 OH), 3.97 (m, 1H, H-34), 3.83 (d, $^3J_{3,2/4}$ = 9.2 Hz, 1H, H-3), 3.74 (m, 1H, H-25), 3.54 – 3.40 (br m + H_2O, H-5 + H-11), 3.28 (m, 1H, H-21), 3.20 (s, 3H, H-31), 3.07 (m, 1H, H-22), 2.91 (dd, $^3J_{33,34}$ = 9.4 Hz, $^3J_{33,33\text{-OH}}$ = 7.7 Hz, 1H, H-33), 2.79 (m, 1H, H-2), 2.67 (s, 6H, H-23), 2.65 (m, 3H, H-8 + H-10 + N-OH), 2.27 (d, $^2J_{29a,29b}$= 15.0 Hz, 1H, H-29a), 1.99 – 1.88 (m, 2H, H-4 + H-24a), 1.81 (m, 1H, H-14a), 1.54 – 1.47 (m, 2H, H-29b + H-7a), 1.44 – 1.34 (m, 3H, H-14b +H-24b +H-7b), 1.33 (s, 3H, H-19), 1.15 (d, $^3J_{35,34}$ = 6.1 Hz, 3H, H-35), 1.14 – 1.12 (m, 6H, H-26 + H-32), 1.10 – 1.07 (m, 9H, H-17 + H-18 + H-36), 1.02 – 0.99 (m, 6H, H-16 + H-27), 0.74 (t, $^3J_{15,14a}$ = $^3J_{15,14b}$ = 7.4 Hz, 3H, H-15); ^{13}C NMR (151 MHz, d_6-DMSO) δ 174.79 (C, C-1), 169.15 (C, C-9), 101.32 (CH, C-20), 95.68 (CH, C-28), 83.08

(CH, C-5), 78.48 (CH, C-3), 77.29 (CH, C-33), 76.11 (CH, C-13), 74.22 (C, C-6 + C-12), 72.73 (C, C-30), 70.37 (CH, C-11), 69.03 (CH, C-21), 66.35 (CH, C-25), 65.05 (CH, C-34), 64.81 (CH, C-22), 48.98 (CH_3, C-31), 44.30 (CH, C-2), 38.49 (CH, C-4), 37.54 (CH_2, C-7), 34.86 (CH_2, C-29), 30.02 (CH_2, C-24), 26.63 (CH_3, C-19), 25.15 (CH, C-8 + C-10), 21.14 (CH_3, C-26), 20.96 (CH_2, C-14), 20.91 (CH_3, C-32), 18.84 (CH_3, C-23), 18.63 (CH_3, C-35), 17.15 (CH_3, C-16), 16.10 (CH_3, C-36), 14.71 (CH_3, C-17 + C-18), 10.67 (CH_3, C-15), 9.26 (CH_3, C-27).

Roxithromycin-C$_4$-N$_3$ ***6***

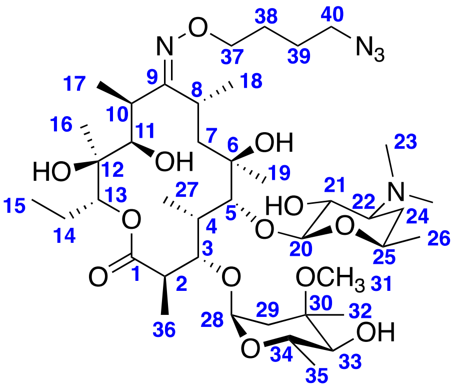

Erythromycin-9-oxime **5** (5.206 g, 6.960 mmol) was suspended in 100 mL Et_2O, then NaH (1.435 g, 60% dispersion in mineral oil, 35.88 mmol) was added. 1-Bromo-4-chlorobutane was then added (4.000 mL, 35.00 mmol), and the white suspension was refluxed overnight. The reaction was then cooled, and diluted with Et_2O and water. The layers were separated, and the aqueous phase was neutralised with 1 M HCl. The organic phase was remixed with the neutralised aqueous. The layers were again separated, and the aqueous phase washed with Et_2O. The combined organic phases were washed with water, then dried with Na_2SO_4, filtered, and concentrated under reduced pressure. The colourless oil was dissolved in 25 mL DMF, and sodium azide (1.517 g, 23.34 mmol) was added. The reaction was heated at 100 °C overnight, then cooled and diluted with Et_2O and H_2O. The layers were separated, and the aqueous phase washed three times with Et_2O. The combined organic phase was washed twice with brine, then dried, filtered, and concentrated under reduced pressure to give an orange solid. Crude product was purified by silica gel chromatography (10% NEt_3 in toluene) to give **Roxi-C$_4$-N$_3$ 6** as an off-white solid (1.080 g, 1.253 mmol, 18%). LCMS: R_t = 3.38 min, C4T07, $[M+H]^+$ = 846.5, purity >99% (210 nm); HRMS (ESI, +) $C_{41}H_{75}N_5O_{13}$ $[M]^+$ calculated 845.5361, observed 845.5385, error 2.83 ppm; ^{1}H NMR (600 MHz, CD_3OD) δ 5.22 (d, $^3J_{13,14}$ = 11.1 Hz, 1H, H-13), 4.93 (d, J = 5.1 Hz, 1H, H-28), 4.59 (d, $^3J_{20,21}$ = 7.0 Hz, 1H, H-20), 4.13 (m, 1H, H-34), 4.07 (m, 2H, H-37), 3.94 (d, $^3J_{3,2}$ = 9.5 Hz, 1H, H-3), 3.81 (m, 1H, H-25), 3.75 (s, 1H, H-11), 3.55 (d, $^3J_{5,4}$ = 7.0 Hz, 1H, H-5), 3.36 (m, 3H, H-40 + H-21), 3.34 (s, 3H, H-31), 3.23 (m, 1H, H-22), 3.07 (d, $^3J_{33,34}$ = 9.4 Hz, 1H, H-33), 2.94 (pent, $^3J_{2,36}$ = $^3J_{2,3}$ = 8.2 Hz, 1H, H-2), 2.74 (m, 2H, H-8 + H-10), 2.70 (s, 6H, H-23), 2.45 (d, $^2J_{29a,29b}$ = 15.2 Hz, 1H, H-29a), 2.05 (m, 1H, H-4), 1.92 (m, 2H, H-14a + H-24a), 1.79 –

1.67 (m, 4H, H-38 + H-39), 1.61 (m, 3H, H-29b + H-7), 1.50 (m, 1H, H-14a), 1.45 (s, 3H, H-19), 1.40 (d, $^2J_{24b,24a}$ = 12.1 Hz, 1H, H-24b) 1.29 (d, $^3J_{35,2}$ = 6.2 Hz, 3H, H-35), 1.25 (m, 6H, H-32 + H-26), 1.22 (d, $^3J_{36,2}$ = 7.1 Hz, 3H, H-36), 1.19 (d, $^3J_{17,10}$ = 7.0 Hz, 3H, H-17), 1.16 (s, 3H, H-16), 1.11 (d, $^3J_{27,4}$ = 7.5 Hz, 3H, H-27), 1.05 (m, 3H, H-18), 0.86 (t, $^3J_{15,14}$ = 7.4 Hz, 3H, H-15); ^{13}C NMR (151 MHz, CD_3OD) δ 177.35 (C, C-1), 171.86 (C, C-9), 103.38 (CH, C-20), 97.84 (CH, C-28), 85.16 (CH, C-5), 81.10 (CH, C-3), 78.95 (CH, C-33), 78.25 (CH, C-13), 75.90 (2 x C, C-6 +C-12), 74.33 (C, C-30), 74.00 (CH_2, C-37), 71.74 (CH, C-11), 71.30 (CH, C-21), 68.36 (CH, C-25), 66.70 (CH, C-34), 66.24 (CH, C-22), 52.34 (CH_2, C-40), 49.99 (CH, C-31), 46.30 (CH, C-2), 40.53 (CH, C-4), 39.88 (CH_3, C-23), 38.95 (CH_2, C-7), 36.05 (CH_2, C-29), 34.50 (2 x CH, H-8 + C-10), 31.25 (CH_2, C-24), 27.67 (CH_3, C-23), 26.79 (CH_2, C-38 +C-39), 22.38 (CH_2, C-14), 21.78 (CH_3, C-32/26), 21.52 (CH_3, C-32/26), 19.21 (CH_3, C-35/18), 19.15(CH_3, C-35/18), 17.23 (CH_3, C-16), 16.54 (CH_3, C-36), 15.07 (CH_3, C-17), 11.12 (CH_3, C-15), 10.11 (CH_3, C-27).

Roxithromycin-C$_4$-Tz-NBD **9**

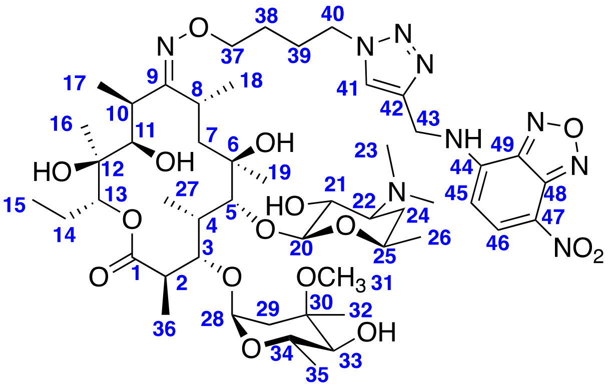

NBD-alkyne **7** was prepared as in Phetsang *et al.*[2] from 4-chloro-7-nitro-benzofuran and propargylamine.

Following methodology from Cochrane *et al.*[3], Roxi-C$_4$-N$_3$ **6** (101.3 mg, 0.1175 mmol) was suspended in 3.0 mL each ^{t}BuOH and H_2O. NBD-alkyne (76.90 mg, 0.3528 mmol) was added, and the orange suspension was heated to 50 °C. To the dark orange solution, copper sulfate (0.7100 mL, 100.0 mM in water, 0.07100 mmol) was added, followed by ascorbic acid (0.5700 mL, 500.0 mM in water, 0.2850 mmol). The reaction was stirred for 1 h, then cooled and diluted in H_2O/Et_2O. The layers were separated, and the aqueous phase washed with Et_2O. The combined organic phases were washed with water, then dried over Na_2SO_4, filtered, and concentrated under reduced pressure. The crude product was purified by MPLC (0 – 100% MeOH in H_2O). This yielded pure **Roxi-C$_4$-Tz-NBD 9** as an orange solid after lyophilisation (47.50 mg, 0.04348 mmol, 37%). LCMS: R_t = 3.71 min, C4T07, $[M]^+$ = 1064.5, purity >96% (210 nm); ^{1}H NMR (600 MHz, d_6-DMSO) δ 8.50 (d, $^3J_{46,45}$ = 9.2 Hz, 1H, H-46), 8.11 (s, 1H, H-41), 6.51 (d, $^3J_{45,46}$ = 8.9 Hz, 1H, H-45), 5.11 (dd,

$^3J_{13,14a}$ = 10.9, $^3J_{13,14b}$ = 2.4 Hz, 1H, H-13), 4.75 (s, 1H, 6-OH), 4.73 (d, $^3J_{28,29}$ = 5.0 Hz, 1H, H-28), 4.41 (d, $^3J_{20,21}$ = 7.4 Hz, 1H, H-20), 4.36 (m, 2H, H-40), 4.27 (d, $^3J_{33OH,33}$ = 7.7 Hz, 1H, 33-OH), 4.00 (m, 1H, H-34), 3.93 (t, $^3J_{37,38}$ = 6.4 Hz, 2H, H-37), 3.89 (m, 1H, H-3), 3.67 (m, 1H, H-25 + H-10 + H-8), 3.61 (s, 1H, H-11), 3.41 (d, $^3J_{5,4}$ = 7.0 Hz, 1H, H-5), 3.31 (CD_3OD + H-23 + H-7), 3.20 (s, 3H, H-31), 3.09 (m, 1H, H-21), 2.90 (m, 1H, H-33), 2.76 (t, $^3J_{2,36}$ = 8.1 Hz, 1H, H-2), 2.63 (d, $^3J_{10,18}$ = 8.3 Hz, 1H, H-10), 2.38 (m 1H, H-25), 2.26 (d, $^2J_{29a,29b}$= 14.8 Hz, H-29a), 1.86 (m, 3H, H-4 + H-39), 1.82 (m, 1H, H-14a), 1.66 (m, 1H, H-24a), 1.53 (m, 3H, H-29b + 38), 1.36 (m, 1H, H-14b) 1.28 (s, 3H,H-19), 1.17 (m, 1H, H-24b), 1.15 (m, 3H, H-35), 1.13 (s, 3H, H-32), 1.08 (m, 6H, H-36 + H-26), 1.03 (d, $^3J_{17,10}$= 7.0 Hz, 3H, H-17), 0.98 (m, 9H, H-27 + H-18 + H-16), 0.74 (t, $^3J_{15,14}$ = 7.4 Hz, 3H, H-15); ^{13}C NMR (151 MHz, d_6-DMSO) δ 175.02 (C, C-1), 169.68 (C, C-9), 144.77 (C, C-44/48/49), 144.58 (C, C-44/48/49), 144.14 (C, C-44/48/49), 142.52 (C, C-42), 137.71 (CH, C-46), 123.33 (CH, C-41), 121.35 (C, C-47), 101.74 (CH, C-20), 99.89 (CH, C-45), 95.46 (CH, C-28), 82.90 (CH, C-5), 78.31 (CH, C-3), 77.37 (CH, C-33), 75.95 (CH, C-13), 74.50 (C, C-12), 73.91 (C, C-6), 72.65 (C, C-30), 71.89 (CH_2, C-37), 70.09 (CH, C-21), 69.69 (CH, C-11), 66.74 (CH, C-25 +H-8 + H-10), 64.90 (CH, C-34), 64.64 (CH, C-10), 49.18 (CH_2, C-40), 48.88 (CH_3, C-31), 44.31 (CH, C-2), 40.00 (C-23 + C-7), 39.87 (CH, C-25), 38.71 (CH, C-4), 34.82 (CH_2, C-29), 29.84 (CH_2, C-24), 26.46 (CH_2, C-39), 26.17 (CH_3, C-19), 25.54 (CH_2, C-38), 21.32 (CH_3, C-26), 21.01 (CH_2, C-14), 20.92 (CH_3, C-32), 18.83 (CH_3, C-18), 18.58 (CH_3, C-35), 17.01 (CH_3, C-16), 15.84 (CH_3, C-36), 14.71 (CH_3, C-17), 10.66 (CH_3, C-15), 9.11 (CH_3, C-27).

Roxithromycin-C$_4$-Tz-DMACA ***10***

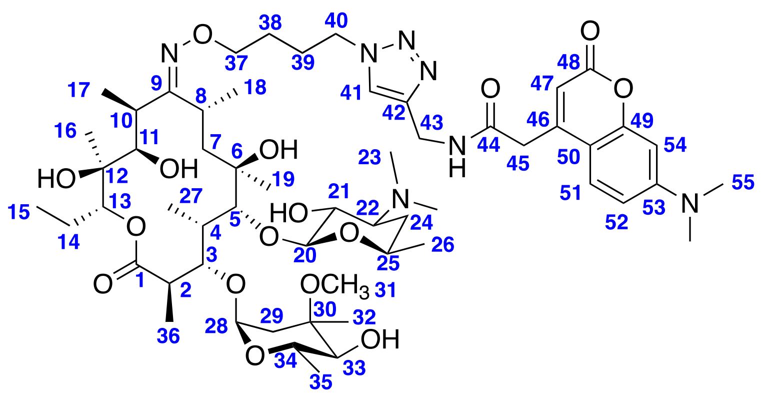

DMACA-alkyne **8** was prepared as in Phetsang *et al.*[2] from 3-(dimethylamino)phenol and propargylamine, via the ethyl ester.

Following methodology from Cochrane *et al.*[3], Roxi-C$_4$-N$_3$ **6** (52.3 mg, 0.0619 mmol) was suspended in 1.5 mL each ^{t}BuOH and H_2O. DMACA-alkyne (22.5 mg, 0.0792 mmol) was added, and the yellow suspension was heated to 50 °C. To the yellow suspension, copper sulfate (0.370 mL, 100 mM in water, 0.0370 mmol) was added,

followed by ascorbic acid (0.300 mL, 500 mM in water, 0.149 mmol). The reaction was stirred for 1.5 h, then 1 mL DMF was added to enhance solubility. After 3 h, additional portions of copper sulfate and ascorbic acid were added, then finally after 3.5 h, the reaction was cooled and diluted in H_2O/Et_2O. The layers were separated, and the insoluble product was filtered off. Crude product was purified by silica gel chromatography (5 – 10% TEA in DCM) to give **Roxi-C$_4$-Tz-DMACA 10** as an off-white solid (25.4 mg, 0.0223 mmol, 36%). LCMS: R_t = 3.62 min, C4T07, $[M/2+H]^+$ = 565.9, purity >97% (210 nm); HRMS (ESI, +) $C_{57}H_{93}N_7O_{16}$ $[M/2+2H]^{2+}$calculated: 565.8334, observed: 565.8340, error -1.1 ppm; ^{1}H NMR (600 MHz, CD_3OD) δ 7.84 (s, 1H, H-41), 7.55 (d, $^3J_{51,52}$ = 9.0 Hz, 1H, H-51), 6.73 (dd, $^3J_{52,51}$ = 9.0, $^4J_{52,54}$ = 2.6 Hz, 1H, H-52), 6.55 (d, $^4J_{54,52}$ = 2.5 Hz, 1H, H-54), 6.04 (s, 1H, H-47), 5.22 (m, 1H, H-13), 4.91 (d, $^3J_{28,29}$ = 5.0 Hz, 1H, H-28), 4.61 (d, $^3J_{20,21}$ = 6.5 Hz, 1H, H-20), 4.47 (s, 2H, H-43), 4.42 (t, $^3J_{40,39}$ = 7.0 Hz, 2H, H-40), 4.10 (m, 1H, H-34), 4.05 – 3.97 (m, 2H, H-37), 3.89 (d, $^3J_{3,2}$ = 9.5 Hz, 1H, H-3), 3.84 (m, 1H, H-25), 3.73 (s, 1H, H-11), 3.53 (d, $^3J_{5,4}$ = 6.8 Hz, 1H, H-5), 3.44 – 3.39 (m, 2H, H-21 + H-22), 3.33 (s, 3H, H-31), 3.19 (m, 2H, H-45), 3.08 (s, 6H, H-55), 3.06 (d, $^3J_{33,34}$ = 9.5 Hz, 1H, H-33), 2.93 (m, 1H, H-2), 2.83 (s, 6H, H-23), 2.72 (m, 2H, H-8+10), 2.43 (d, $^2J_{29a,29b}$ = 15.2 Hz, 1H, H-29a), 2.05 (m, 1H, H-4), 2.01 – 1.94 (m, 3H, H-24a + H-39), 1.90 (m, 1H, H-14a), 1.66 – 1.56 (m, 4H, H-29b + H-7a + H-38), 1.54 – 1.45 (m, 3H, H-14b + H-7b +H-24b), 1.42 (s, 3H, H-19), 1.28 – 1.23 (m, 9H, H-35 + H-32 +H-26), 1.20 (d, $^3J_{36,2}$ = 7.1 Hz, 3H, H-36), 1.16 (s, 3H, H-17), 1.15 (m, 6H, H-16 + H-18), 1.09 (m, 3H, H-27), 0.85 (t, $^3J_{15,14}$ = 7.4 Hz, 3H, H-15); ^{13}C NMR (151 MHz, CD_3OD) δ 177.23 (C, C-1), 171.92 (C, C-9), 170.96 (C, C-44), 164.22 (C, C-48), 157.17 (C, C-49), 154.80 (C, C-53), 152.74 (C, C-46), 145.92 (C, C-42), 127.02 (CH, C-51), 124.33 (CH, C-41), 110.56 (CH, C-52), 110.47 (CH, C-47), 109.80 (C, C-50), 103.04 (CH, C-20), 98.74 (CH, C-54), 97.78 (CH, C-28), 85.31 (CH, C-5), 81.06 (CH, C-3), 78.81 (CH, C-33), 78.22 (CH, C-13), 75.83 (2 x C, C-6 + C-12), 74.35 (C, C-30), 73.59 (CH_2, C-37), 71.75 (CH, C-11), 70.66 (CH, C-21), 68.08 (CH, C-25), 66.70 (CH, C-34/22), 66.64 (CH, C-34/22), 51.15 (CH_2, C-40), 49.97 (CH_3, C-31), 47.88 (CH_2, C-45), 46.27 (CH, C-2), 40.49 (CH, C-4), 40.46 (CH_3, C-23), 40.28 (CH_3, C-55), 38.76 (CH_2, C-7), 36.00 (CH_2, C-43), 35.92 (CH_2, C-29), 34.33 (CH, C-8 + C-10), 31.05 (CH_2, C-24), 30.71 (CH_2, C-38), 27.99 (CH_2, 39), 27.62 (CH_3, C-19), 27.16 (CH_2, C-38), 22.36 (CH_2, C-14), 21.75 (CH_3, C-35/C-32), 21.52 (CH_3, C-35/C-32), 19.17 (CH_3, C-26), 17.25 (2 x CH_3, C-16 + C-18), 16.62 (CH_3, C-36), 11.14 (CH_3, C-15), 10.11 (CH_3, C-27).

NMR Spectra

MRLSPhD4073.1.fid

^{1}H NMR (600 MHz, DMSO) δ 5.89, 5.13, 4.74, 4.46, 4.36, 4.09, 4.03, 3.97, 3.83, 3.74, 3.52, 3.47, 3.46, 3.29, 3.20, 3.07, 2.91, 2.78, 2.67, 2.28, 1.92, 1.81, 1.52, 1.40, 1.33, 1.16, 1.13, 1.08, 1.01, 0.74.

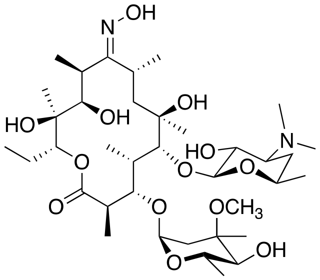

Erythromycin-9-oxime **5**

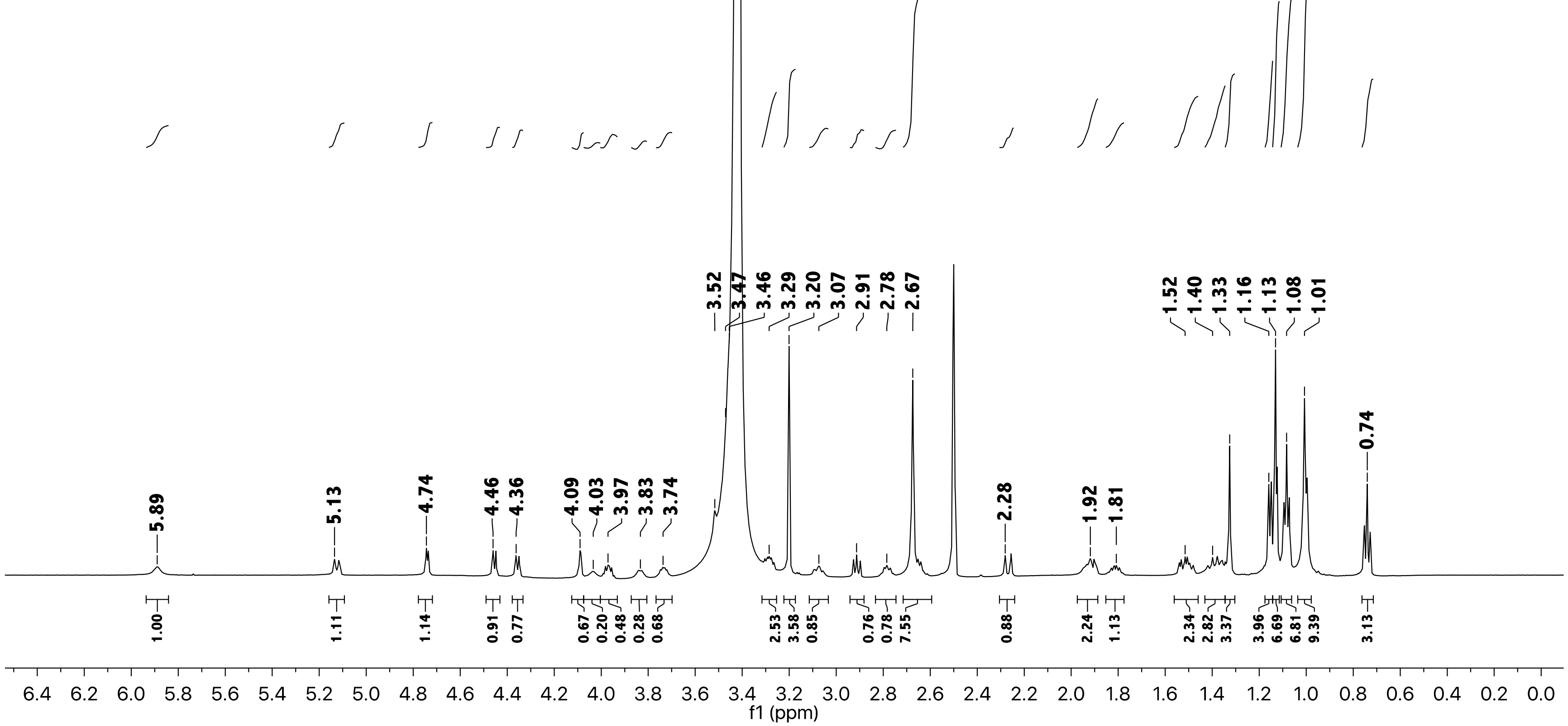

MRLSPhD4073.3.fid

^{13}C NMR (151 MHz, DMSO) δ 174.77, 169.15, 101.32, 95.68, 83.07, 78.48, 77.29, 76.11, 74.22, 72.73, 70.37, 69.03, 66.34, 65.05, 64.80, 48.98, 44.29, 38.49, 37.58, 34.86, 30.01, 26.63, 25.15, 21.14, 20.92, 18.84, 18.63, 17.15, 16.10, 14.71, 10.67, 9.26.

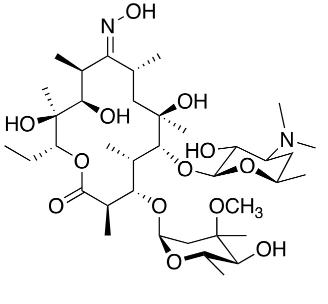

Erythromycin-9-oxime **5**

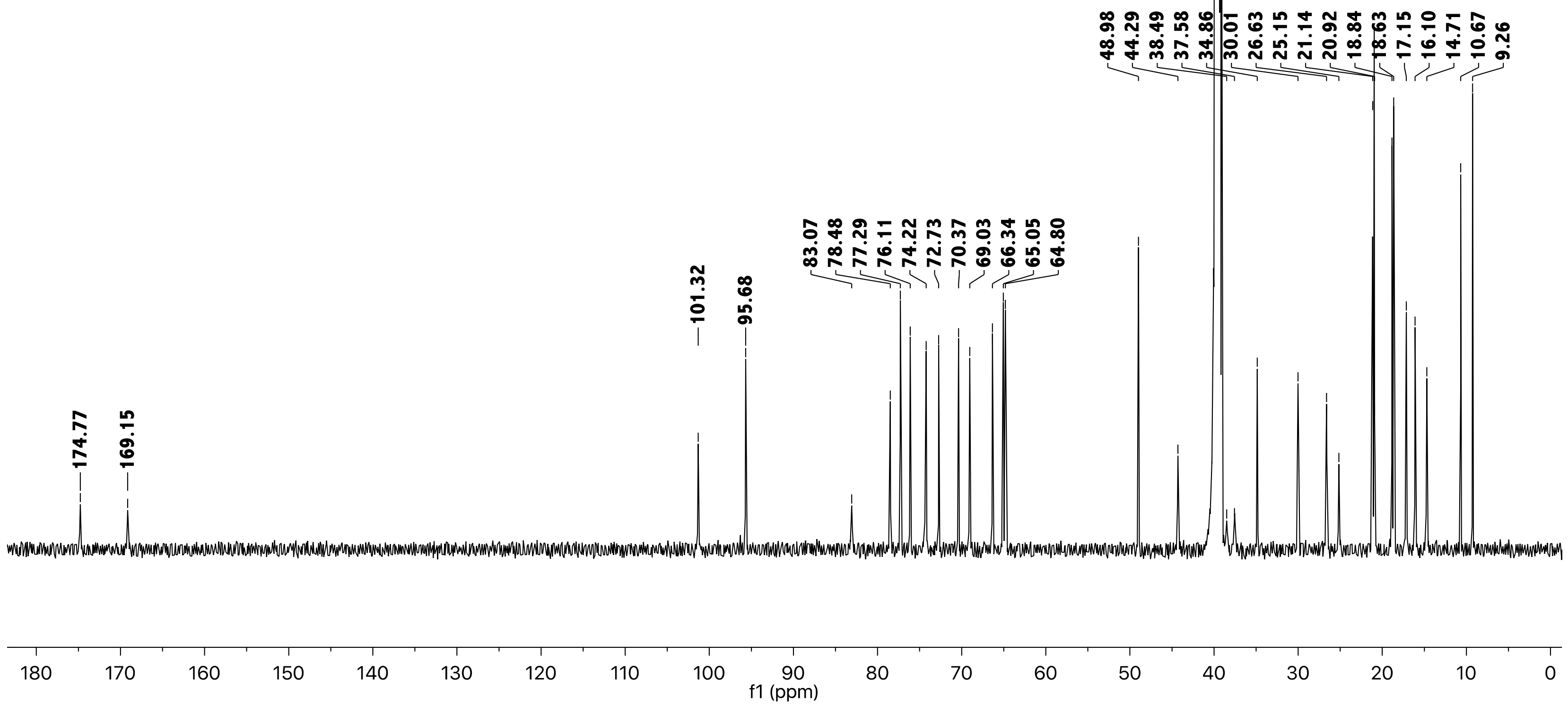

MRLSPhD03003 1.fid

Roxi-C_4-N_3 **6**

^{1}H NMR (600 MHz, MeOD) δ 5.22, 4.92, 4.59, 4.13, 4.06, 3.94, 3.80, 3.75, 3.55, 3.36, 3.34, 3.22, 3.07, 2.93, 2.74, 2.69, 2.46, 2.04, 1.92, 1.73, 1.61, 1.50, 1.45, 1.40, 1.28, 1.25, 1.21, 1.18, 1.15, 1.10, 1.04, 0.86.

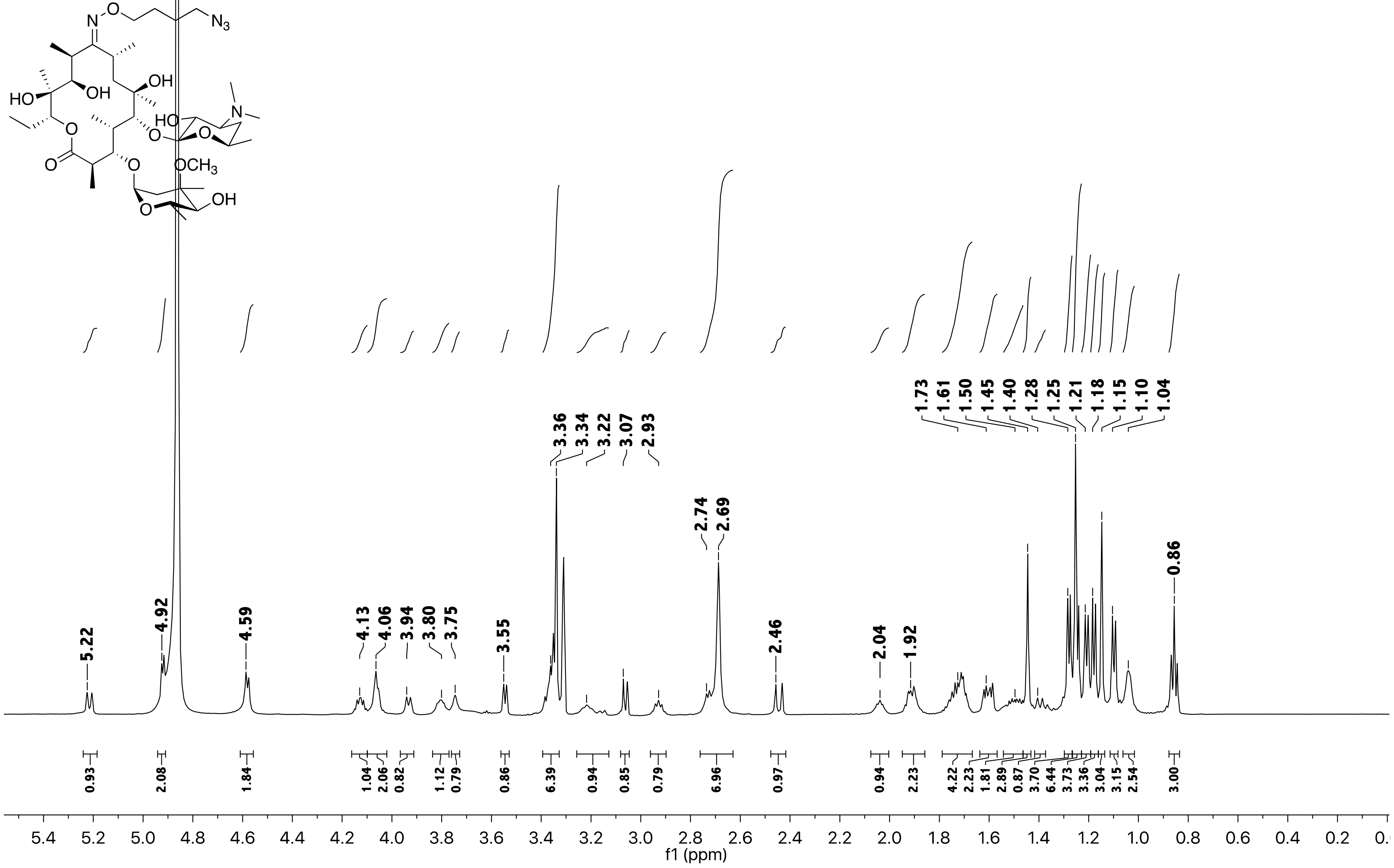

MRLSPhD03003.3.fid

^{13}C NMR (151 MHz, MeOD) δ 177.34, 171.86, 103.38, 97.84, 85.16, 81.10, 78.95, 78.25, 75.90, 74.33, 74.00, 71.73, 71.30, 68.36, 66.70, 66.23, 52.34, 49.99, 46.30, 40.53, 39.88, 38.95, 36.05, 34.47, 31.25, 27.67, 27.52, 27.19, 26.79, 22.38, 21.77, 21.52, 19.21, 19.15, 17.23, 16.54, 15.07, 11.12, 10.11.

Roxi-C_4-N_3 **6**

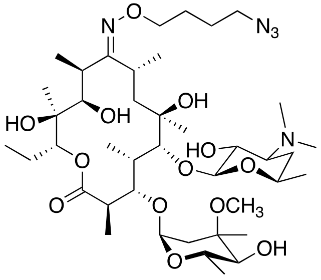

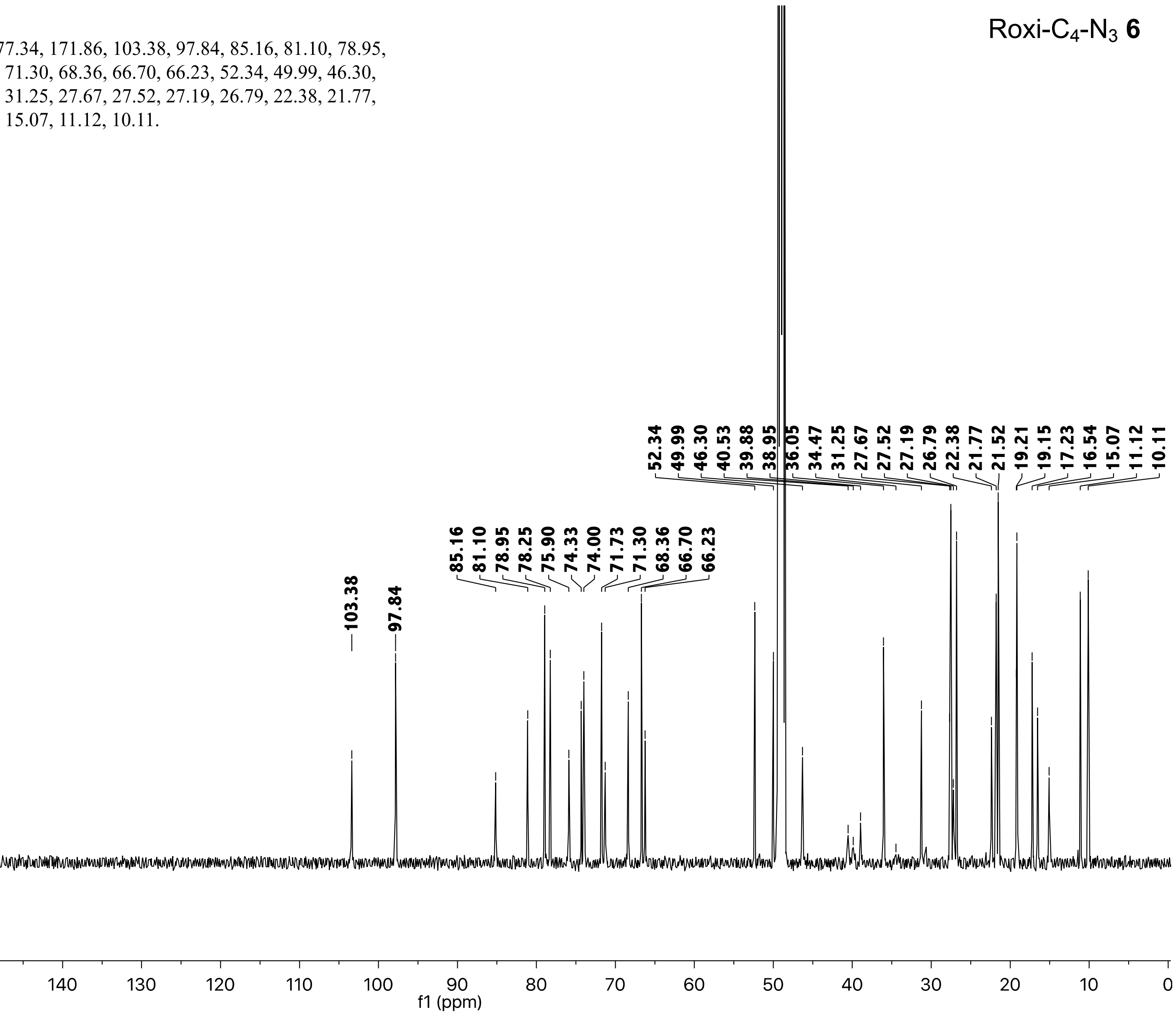

MRLSPhD03024.1.fid

Roxi-C_4-Tz-NBD **9**

^{1}H NMR (600 MHz, DMSO) δ 8.52, 8.11, 6.52, 5.11, 4.73, 4.41, 4.36, 4.28, 4.00, 3.93, 3.90, 3.67, 3.60, 3.42, 3.33, 3.29, 3.20, 3.09, 2.90, 2.76, 2.63, 2.38, 2.28, 1.86, 1.81, 1.66, 1.53, 1.45, 1.35, 1.28, 1.18, 1.16, 1.13, 1.08, 1.04, 0.99, 0.74.

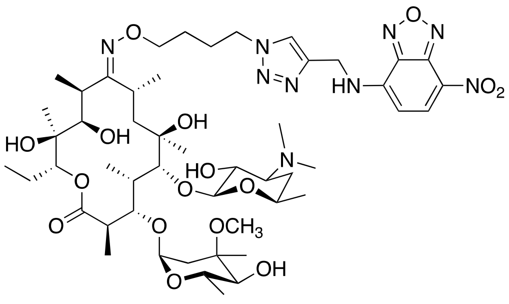

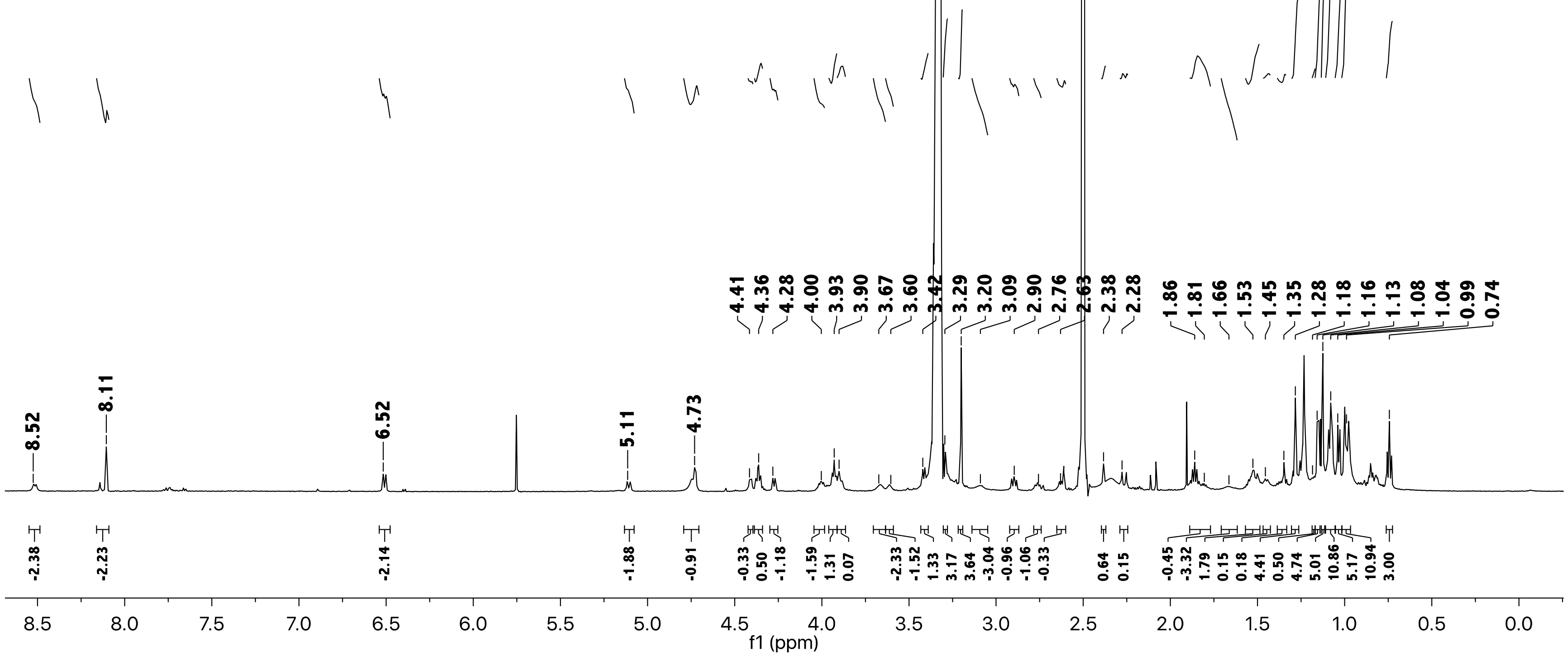

MRLSPhD03024.5.fid

^{13}C NMR (151 MHz, DMSO) δ 175.02, 169.68, 144.78, 144.59, 144.14, 142.52, 137.71, 123.33, 121.35, 101.74, 99.90, 95.47, 82.90, 78.31, 77.37, 75.95, 74.29, 73.91, 72.65, 71.90, 70.10, 69.69, 66.74, 64.91, 64.64, 49.19, 48.88, 44.32, 39.52, 38.71, 34.82, 29.84, 26.46, 26.18, 25.54, 21.32, 21.01, 20.92, 18.83, 18.58, 17.01, 15.84, 14.71, 10.66, 9.11.

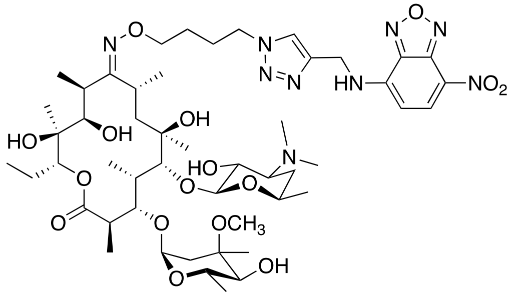

Roxi-C_4-Tz-NBD **9**

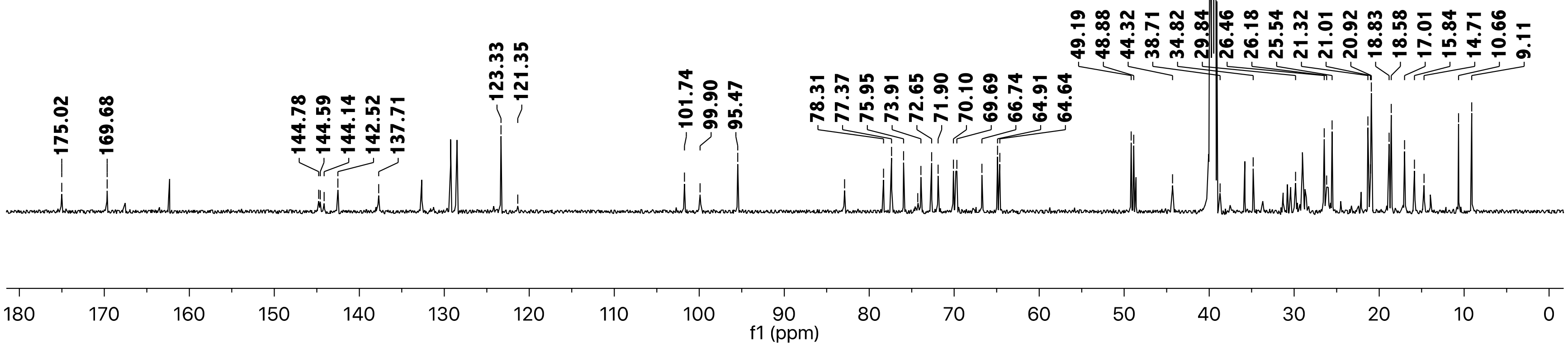

roxi-dmaca.1.fid

Roxi-C_4-Tz-DMACA **10**

^{1}H NMR (600 MHz, MeOD) δ 7.84, 7.55, 6.74, 6.55, 6.04, 5.23, 5.21, 4.91, 4.62, 4.47, 4.42, 4.10, 4.02, 3.90, 3.83, 3.73, 3.53, 3.42, 3.33, 3.21, 3.08, 3.05, 2.93, 2.83, 2.72, 2.44, 1.98, 1.90, 1.64, 1.59, 1.48, 1.42, 1.27, 1.26, 1.25, 1.21, 1.16, 1.15, 1.10, 0.85.

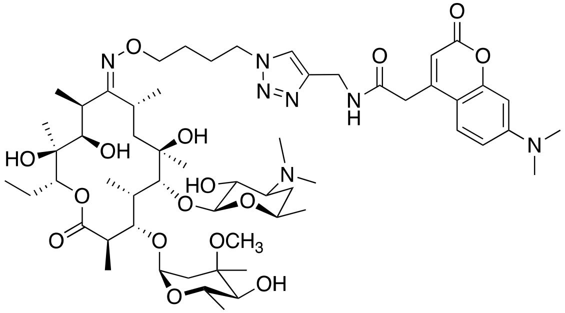

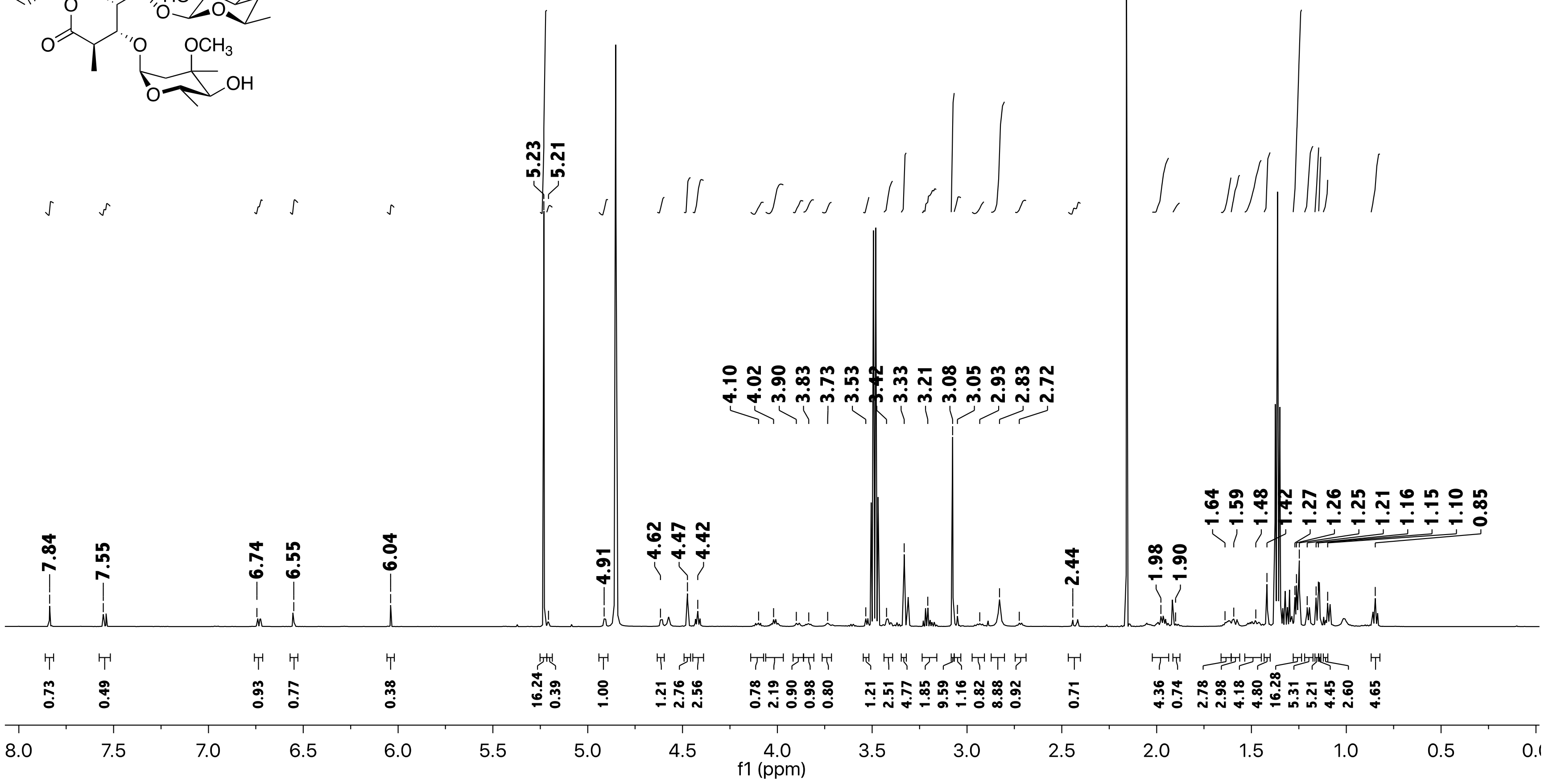

Minimum Inhibition Concentration Determination

Glycerol stocks of bacterial strains were streaked on LB or TSA (with 5% defibrillated sheep's blood) agar and grown overnight at 37 °C. Some species were incubated with 5% CO_2 throughout. Single colonies were then picked and cultured overnight in CAMHB or BHI at 37 °C, then diluted ~40-fold and grown to $OD_{600} = 0.4 – 0.8$. Stock solutions of each compound were prepared at 1.28 mg/mL in sterile water, and 10 µL were added to the first column of a 96-well plate. 90 µL CAMHB or BHI was added to the first column, and 50 µL to all other wells. Serial 2-fold dilution was then carried out across the plate. The mid-log phase cultures were diluted to ~10^6 colony forming units (cfu)/mL and 50 µL was added to wells, to give a final concentration of ~$5x10^5$ cfu/mL. Plates were covered and incubated at 37 °C for 18 – 24 h then the MICs were determined visually, with the MIC being the lowest concentration well with no visible growth.

Microscopy Preparation

Single colonies were picked and cultured overnight as for MIC, then diluted ~40-fold and grown to OD_{600} = 0.4 – 0.8. Cultures were centrifuged at 18,000 x g and broth decanted. The pellets were suspended in Hanks' Balance Salt Solution (HBSS), centrifuged, and the liquid decanted. Pellets were then resuspended in 500 µL of HBSS containing 10 µM roxi-C_4-Tz-DMACA **10** and in appropriate samples, 10 µM CCCP. The samples were incubated at room temperature in the dark for 30 min, then centrifuged for 3 min and 18,000 x g. The media was decanted and the bacteria washed with 1000 µL of HBSS. Next, the bacteria were resuspended in 500 µL of 5 µM Syto-9 in HBSS, then incubated at room temperature in the dark for 20 min. Bacteria were then centrifuged, the media decanted, then resuspended in 500 µL of 5 µg/mL FM4-64FX in HBSS. The samples were incubated for 5 min on ice in the dark, then centrifuged and decanted. A final washing was then carried out to yield tri-labelled, washed bacteria. The pellets were resuspended in 10 µL of HBSS, and 2 µL of this was spread on a microscope slip, then let to dry. This was then mounted with ~15 µL of Vectashield, and the edges sealed with clear nail polish.

Additional Microscopy Images

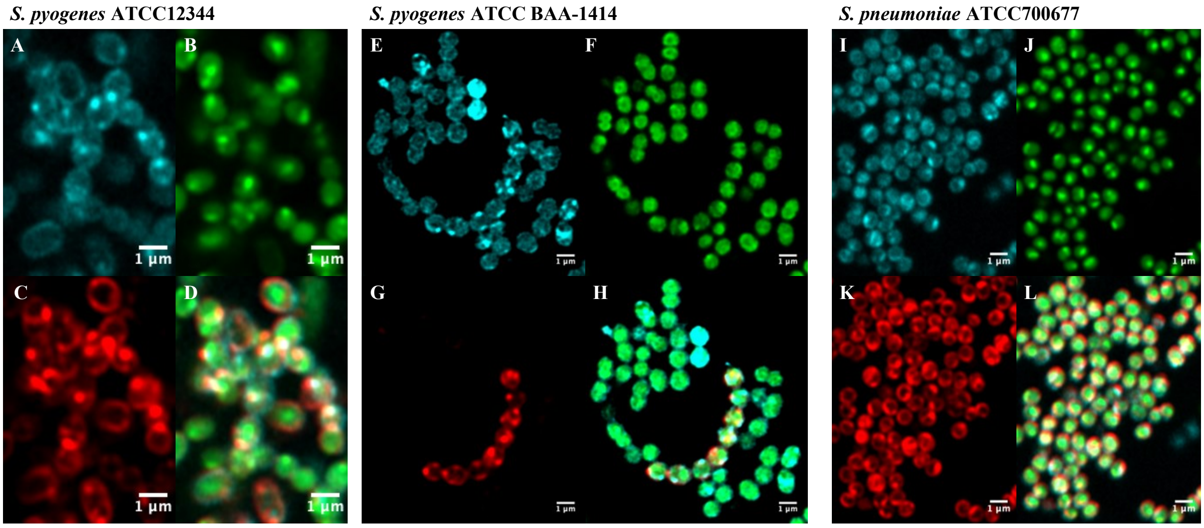

Figure S1: Confocal microscopic images of live **(A-D)** susceptible *S. pyogenes* (ATCC 12344), **(E-H)** resistant *S. pyogenes* (ATCC BAA-1414), and **(I-L)** resistant *S. pneumoniae* (ATCC 700677) labelled with **A,E,I**: roxi-C_4-Tz-DMACA **10** (10 μM); **B,F,J**: Syto-9 (green nucleic acid dye, 5 μM); **C,G,K**: FM4-64FX (red membrane dye, 5 μg/mL); and **D,H,L**: overlaid.

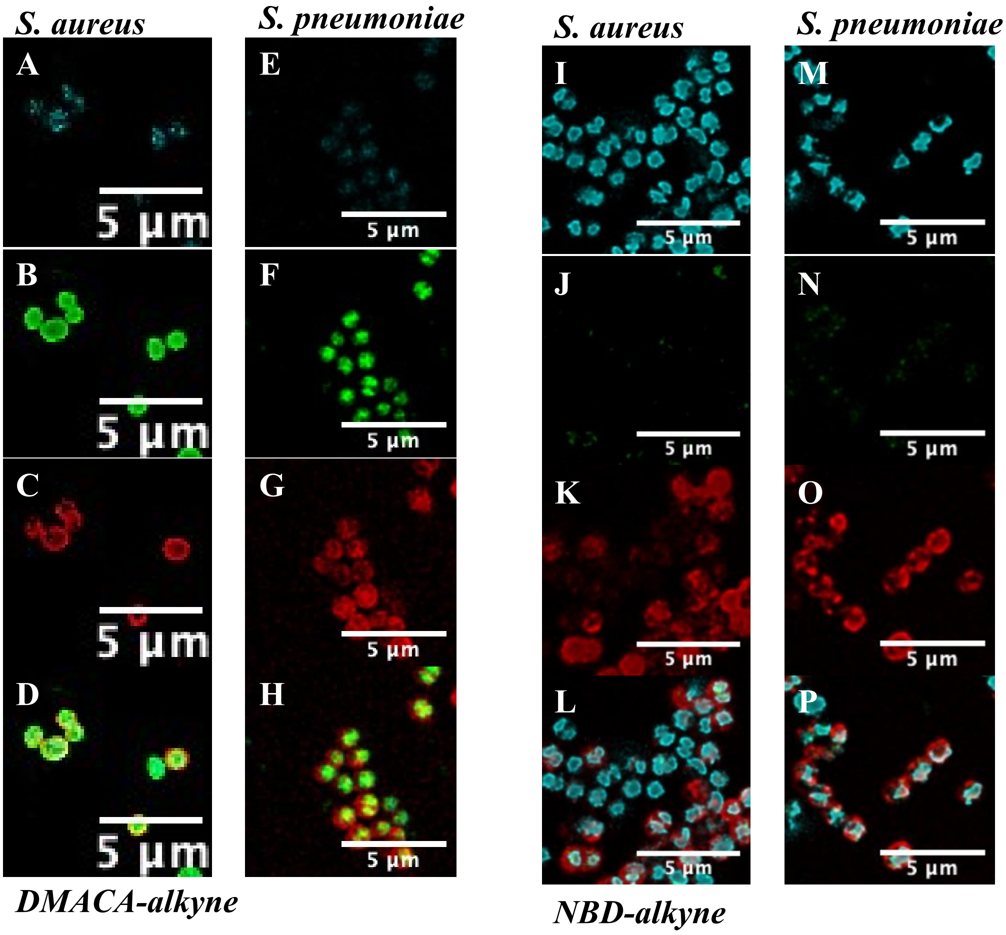

Figure S2: Confocal microscopic images of live susceptible *S. aureus* (ATCC 25923, left hand columns), and *S. pneumoniae* (ATCC 33400, right hand columns) with **A,E**: DMACA-alkyne **8** (10 μM) or **J,N:** NBD-alkyne **7** (10 μM); **B,F**: Syto-9 (green nucleic acid dye, 5 μM) or **I,M:** Hoechst-33342 (blue nucleic acid dye, 5 μg/mL); **C,G,K,O**: FM4-64FX (red membrane dye, 5 μg/mL); and **D,H,L,P**: overlaid.

Microfluidics Methodology

Microbiology

LB medium (10 g/L tryptone, 5 g/L yeast extract, and 10 g/L NaCl) was used for planktonic growth. M9 minimal media was purchased from Sigma. *E. coli* BW25113 was purchased from Dharmacon (GE Healthcare). Overnight cultures were prepared by picking a single colony of *E. coli* BW25113 from a streak plate and growing it in 200 mL fresh LB medium in a shaking incubator at 200 rpm and 37 °C for 17 h.

Fabrication of the microfluidics devices

The mould for the mother machine microfluidic device[4] was obtained by pouring epoxy onto a polydimethylsiloxane (PDMS, Dow Corning) replica of the original mould containing 12 independent microfluidic chips (kindly provided by S. Jun). Each of these chips was equipped with approximately 6000 lateral microfluidic channels with width and height of approximately 1 μm each and a length of 25 μm. These lateral channels were connected to a main microfluidic chamber that is 25 and 100 μm in height and width, respectively. PDMS replicas of this device were realised as previously described.[5] Briefly, a 9:1 (base:curing agent) PDMS mixture was cast on the mould and cured at 70 °C for 120 min in an oven. The cured PDMS was peeled from the epoxy mould and cut into individual chips. Fluidic accesses were created by using a 0.75 mm biopsy punch (Harris Uni-Core, WPI). The PDMS chip was irreversibly sealed on a glass coverslip by exposing both surfaces to oxygen plasma treatment (10 s exposure to 30 W plasma power, Plasma etcher, Diener, Royal Oak, MI, USA). This treatment temporarily rendered the PDMS and glass hydrophilic, so within 5 min after bonding the chip was filled with 2 μL of a 50 mg/mL bovine serum albumin solution and incubated at 37 °C for 1 h, thus passivating the device's internal surfaces and preventing subsequent cell adhesion.

Microfluidics-microscopy assay

An overnight culture was prepared as described above. Spent LB broth and bacteria were prepared by centrifuging the overnight culture (10 min at 3000 x g and 20 °C). The supernatant was filtered twice (Medical Millex-GS Filter, 0.22 μm, Millipore Corp.) and used to resuspend the bacteria in their spent LB to an optical density of 50 at 595 nm. Bovine serum albumin was added to the bacterial suspension at a concentration of 0.5 mg/mL. A 2 μL aliquot of this suspension was injected in the above described microfluidic device and incubated at 37 °C. The high bacterial concentration favours bacteria entering the narrow lateral channels from the

main microchamber of the mother machine.[4] We found that an incubation time of 20 min allowed filling of the lateral channels with, typically, between one and three bacteria per channel. An average of 60% of lateral channels of the mother machine device were filled with bacteria and 50% of the filled channels contained single bacteria. This facilitated tracking each single bacterium throughout the different phases of our assay. The microfluidic device was completed by the integration of fluorinated ethylene propylene tubing (1/32" × 0.008"). The inlet tubing was connected to a flow-rate measuring device (Flow Unit S, Fluigent, Paris, France) controlling the pressure applied by a computerized pressure-based flow control system (MFCS-4C, Fluigent) on the inlet reservoir feeding the flow rate device itself. This instrumentation was controlled by MAESFLO software (Fluigent). At the end of the 20 min incubation period, the chip was mounted on an inverted microscope (IX73 Olympus, Tokyo, Japan) and the bacteria remaining in the main microchamber of the mother machine were washed into the outlet tubing and reservoir by flowing spent LB at 300 μL/h for 8 minutes. At this point we acquired our first set of images in bright-field and fluorescence mode. Images were collected via a 60×, 1.2 NA objective (UPLSAPO60XW, Olympus) and a sCMOS camera (Zyla 4.2, Andor, Belfast, UK). The region of interest of the camera was adjusted to visualise 23 lateral channels per image. Upon acquiring each bright-field image the microscope was switched to fluorescent mode and FITC filter using custom built Labview software and a fluorescence image was acquired by exposing the bacteria to the blue excitation band of a broad-spectrum LED (CoolLED pE300white, Andover, UK) at 20% of its intensity. These parameters were adjusted in order to maximize the signal to noise ratio. The device was moved by two automated stages (M-545.USC and P-545.3C7, Physik Instrumente, Karlsruhe, Germany, for coarse and fine movements, respectively) to image the next set of lateral channels and these steps were repeated until approximately 100 bacteria were imaged. After acquiring the first set of images, the microfluidic environment was changed by flowing M9 with roxi-C_4-Tz-NBD **9** at 46 μg/mL at 300 μL/h for 8 minutes and then at 100 μL/h for 2 h. Imaging was carried out every 20 min for 6 hours. After 2 h, the probe was removed and LB medium was flowed in the chip at 300 μL/h for 8 minutes and then at 100 μL/h for 4 h. The entire assay was carried out at room temperature.

Image and data analysis

Microfluidics confinement and time-lapse microscopy allow the tracking of each individual bacterium and its eventual progeny throughout the entire assay. Images were processed using ImageJ software. A rectangle was drawn around each bacterium at every time point, obtaining

its width, length and mean fluorescence intensity. The mean fluorescence intensity for each bacterium was normalised by cell size, to account for variations in drug accumulation due to the cell cycle. The background fluorescence (i.e. the fluorescence of extracellular drug) at each time point was calculated; mean fluorescence intensity was measured by drawing a rectangle, of similar dimensions to those drawn around the bacteria and positioned, at the same distance from the main channel, in the nearest empty lateral channel that did not contain bacteria. This mean background fluorescence value was subtracted from each bacterium's fluorescence value at every respective time point.

Efflux Model System Accumulation Assay

mar1 E. coli was kindly provided by Dr Muriel Masi (AG102).[6] Glycerol stocks of bacterial strains were streaked on LB agar and grown overnight at 37 °C. Single colonies were then picked and cultured overnight in LB at 37 °C, then diluted ~40-fold and grown to OD_{600} = 0.4 – 0.6. Cultures were centrifuged at 7,700 x g for 15 min at 20 °C and broth decanted. The pellets were resuspended in PBS for a final OD_{600} of 6, then for each sample, 2.4 mL of the appropriate culture was mixed with CCCP (100 μM in water, final concentration 10 μM) and/or fluorescent probe (500 μM in 10% DMSO, final concentration 50 μM) and/or PBS for a total volume of 3 mL. At each time point, 400 μL was removed and mixed with 600 μL glycine-HCl. The lysed cell cultures were centrifuged at 18,000 x g and the lysate collected. 200 μL of each lysate was added to a black-sided 96-well microplate and the fluorescence signal measured (NBD λ_{ex} = 450 nm, DMACA λ_{ex} = 370 nm). Statistical significance was determined by t-test, using the Holm-Sidak method, with an alpha of 0.05.

Probe Accumulation and Localisation Determination Assay

Workflow

Single colonies were picked and cultured overnight as above, then diluted ~40-fold and grown to OD_{600} = 0.4 – 0.8. Cultures were then centrifuged at 6,000 x g for 15 min at 20 °C, then the media was decanted. Bacterial pellets were resuspended in PBS (50 mL for an original culture volume of 350 mL), then centrifuged again and media decanted. Bacteria were then resuspended in PBS for a final OD_{600} of 6. To each well of a 2 mL-capacity 96-well deep well plate, 1.44 mL of the bacteria was added, followed by 0.36 mL of 5 μM probe solution in PBS (from 5 mM stocks in DMSO), then mixed. The plates were then incubated in the dark at room temperature for 30 min, mixed, then 200 μL was removed to a 96-well black plate (full aliquot,

Figure S3). The deep-well plate was centrifuged at 3,300 x g for 30 min at 4 °C, then 200 μL of the decantate was removed to a 96-well black plate (excess aliquot). The remainder of the media was decanted, then the pellet was resuspended in 500 μL of PBS. The deep-well plate was centrifuged at 3,300 x g for 20 min at 4 °C, then 200 μL of the decantate was removed to a 96-well black plate (wash aliquot) and the remainder of the media was discarded. The washed pellet was resuspended in 400 μL of PBS, then 200 μL of the decantate was removed to a 96-well black plate (total aliquot). To each deep-well, 300 μL of B-PER containing 0.2 mg/mL lysozyme was added, then the wells mixed. The deep-well plate was then frozen at -80 °C (placed in -80 °C freezer for 1 h) and defrosted at 37 °C (placed in 37 °C incubator for 1 h). Next, the defrosted plate was centrifuged at 3,300 x g for 20 min at 4 °C, then 200 μL of the decantate was removed to a 96-well black plate (IF aliquot). Lastly, the pellet was resuspended in 200 μL of PBS, then moved to a 96-well black plate (pellet aliquot). Each set of aliquots on black plate was read on a spectrophotometer as soon as practicable after collection at wavelengths appropriate for each probe, in addition to tryptophan (Trp) fluorescence (Trp λ_{ex} = 280 nm, λ_{em} = 350 nm; DMACA λ_{ex} = 390 nm, λ_{em} = 490 nm; NBD λ_{ex} = 450 nm, λ_{em} = 540 nm).

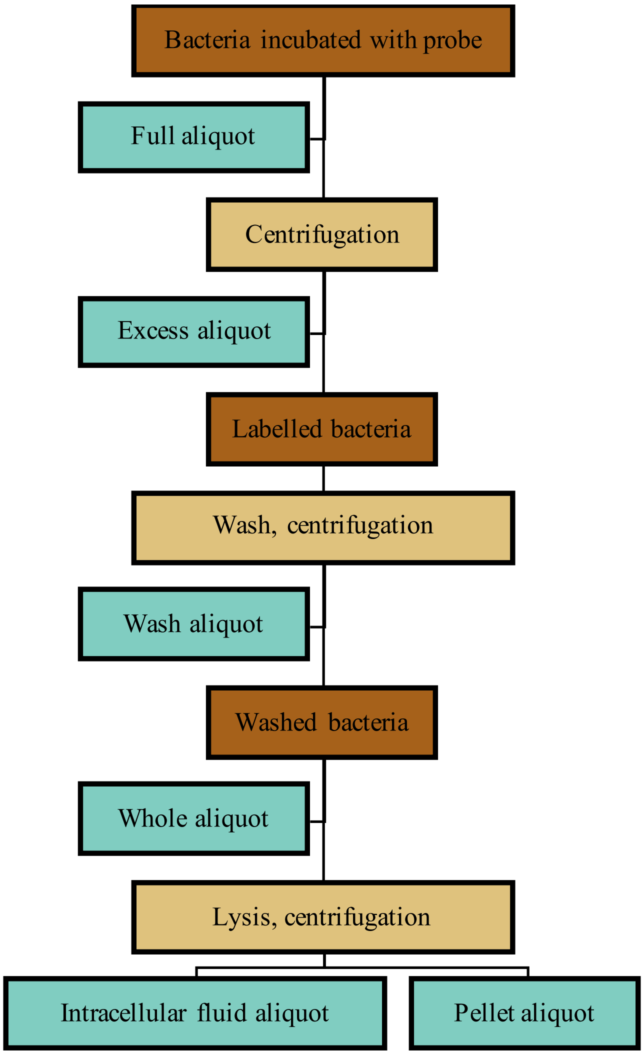

Figure S3: Flowchart of labelling and localisation quantification assay steps

Data Processing

In order to scale the raw values obtained to the total signal, each aliquot was given a multiplication factor comprised of the proportion of total volume each stage (see **Table S1** for details) and the proportion of bacteria remaining at each stage (see **Table S1** for details). This scaling transformed the raw values at each stage to represent the signal that would be present for the initial amount of bacteria. It was assumed that all aliquots are representative of the whole, that no bacteria are lost in the excess or wash stages, and that all bacteria are lysed.

Table S1: Example calculations of raw data scaling

Stage	Proportion of stage (dilution)	Proportion of bacteria left for sampling	Total multiplication factor
**Total**	200/1800 = 0.1111	100%	x 9
**Excess**	200/1600 = 0.125	88.89%	x 9
**Wash**	200/500 = 0.4	88.89%	x 2.8125
**Whole**	200/400 = 0.5	88.89%	x 2.25
**IF**	200/500 = 0.4	44.44%	x 5.625
**Pellet**	200/200 = 1.0	44.44%	x 2.25

After scaling to the whole, signals can then be scaled to the tryptophan signal present, which is used as a measure of the amount of bacteria present. For each stage, the tryptophan signals were averaged, and each individual tryptophan signal divided by the stage average (see **Table S2** for examples). This multiplication factor was then used on the probe fluorescence signals to scale them to the amount of tryptophan in each well.

Table S2: Example calculations for average tryptophan scaling

	**Full Aliquot Example Values**		
	Rep1	*Rep2*	*Rep3*
**Raw Trp**	6876	6793	6751
**Scaled Trp**	61884	61137	60759
**Average Trp**	61260		
**Trp scale factor**	0.9899	1.0020	1.0082
**Raw DMACA**	924	889	931
**Scaled DMACA**	8316	8001	8379
**Scaled+ Trp scaled DMACA**	8370	8062	8263

The next correction that was applied to the data was to use signals gathered from bacteria incubated with roxithromycin (as opposed to the fluorescent probes) to correct for plate fluorescence, reagent fluorescence, bacterial autofluorescence, and any fluorescence associated with the macrolide core. The appropriately scaled signal from the roxi-bacteria was subtracted from the probe signal to give a more accurate representation of the fluorescence originating from the probe alone (**Table S3**).

Table S3: Example calculations of unlabelled antibiotic subtraction

	Full Aliquot Example Values		
	Rep1	*Rep2*	*Rep3*
**Raw DMACA: 10**	924	889	931
**Scaled DMACA: 10**	8316	8001	8379
**Scaled+ Trp scaled DMACA: 10**	8370	8062	8263
**Raw DMACA: 2**	98	96	99
**Scaled DMACA: 2**	882	864	891
**Scaled+ Trp scaled DMACA: 2**	888.7	867.1	881.2
**10 – 2**	7491	7183	7384

Lastly, the impact of assay step-specific reagents on the probe fluorescence was assessed and corrected for. In order to do this, aliquots were taken from negative control set of wells as usual, then added to a black plate and mixed with probes. The fluorescence was compared to the same concentration of probe mixed in plain PBS, then scale factors calculated (**Table S4**). The fluorescence of the alkynes did not undergo any significant changes in any steps' reagents, and the roxithromycin probes were only impacted in the IF and pellet reagents. This is consistent with the lysis reagents (B-PER with lysozyme) being responsible, as this would be present in the bulk or residue in these steps. Determination of the fluorescence in solutions containing only either B-PER or lysozyme (0.2 mg/mL in PBS) showed that the B-PER was the major player in this in this phenomenon. This bias in fluorescence is most likely due to the detergent present in B-PER (proprietary non-ionic detergent in Tris buffer, Thermo-Fisher).

Table S4: Step-specific reagent biases on probe fluorescence

	Aliquot					
Probe	Full	Excess	Wash	Whole	IF	Pellet
10	PBS: 203	PBS: 303	PBS: 163	PBS: 2261	PBS: 275	PBS: 264
	Reagent: 214	Reagent: 320	Reagent: 152	Reagent: 2157	Reagent: 1388	Reagent: 1655
	S.F.: 0.948	S.F.: 0.947	S.F.: 1.07	S.F.: 1.05	S.F.: 0.198	S.F.: 0.160
9	PBS: 88	PBS: 114	PBS: 72	PBS: 802	PBS: 96	PBS: 104
	Reagent: 101	Reagent: 152	Reagent: 70	Reagent: 863	Reagent: 714	Reagent: 839
	S.F.: 0.871	S.F.: 0.750	S.F.: 1.03	S.F.: 0.929	S.F.: 0.134	S.F.: 0.124
8	PBS: 350	PBS: 365	PBS: 414	PBS: 1468	PBS: 578	PBS: 935
	Reagent: 370	Reagent: 397	Reagent: 411	Reagent: 1494	Reagent:442	Reagent: 694
	S.F.: 0.946	S.F.: 0.919	S.F.: 1.01	S.F.: 0.982	S.F.: 1.31	S.F.: 1.34
7	PBS: 697	PBS: 694	PBS: 710	PBS: 2693	PBS: 1028	PBS:1725
	Reagent: 741	Reagent: 738	Reagent: 736	Reagent: 2734	Reagent: 579	Reagent: 1468
	S.F.: 0.941	S.F.: 0.940	S.F.: 0.964	S.F.: 0.985	S.F.: 1.35	S.F.: 1.18

With the impact of reagent on fluorescence quantified, these scale factors were applied to the values previously obtained, to eliminate changes in fluorescence cause by the reagents (**Table S5**).

Table S5: Example calculation of dye scaling

	**Full Aliquot Example Values**		
	Rep1	*Rep2*	*Rep3*
**Raw DMACA: 10**	924	889	931
**Scaled DMACA: 10**	8316	8001	8379
**Scaled+ Trp scaled DMACA: 10**	8370	8062	8263
**10 – 2**	7491	7183	7384
**Dye scale factor**	0.948		
**10 – 2 scaled to dye**	7106	6814	7004

Additional Results and Discussion

Before a more detailed analysis of the data was carried out, a number of interesting phenomena observed in the data were addressed. First, the *erm(B)+ S. pneumoniae* strain used in the assay (ATCC 700677) proved to be very hard to manipulate, due to a propensity to form hydrophobic clots that were not amenable to suspension or transfer. Reduced uptake of probes and controls were observed, and it is hypothesised that this is due to poor penetration. The results obtained using this strain were highly variable due to the inconsistent uptake, hence significant

conclusions about the impact of the *erm(b)* gene cannot be made at this point. In the future, different strains should be investigated in order to properly examine the impact of this important resistance mechanism.

The second set of unexpected results concern the continuity of the aliquots from a given sample. As can be seen in **Figure 7** and **Figure 8**, in both susceptible *S. aureus* and *S. pneumoniae*, the fluorescence in the excess aliquots was consistently higher than that in the full (**Figure S4, A**). This is illogical, given that the full aliquot is the progenitor of the excess aliquot, with the only steps in between being centrifugation and decanting.

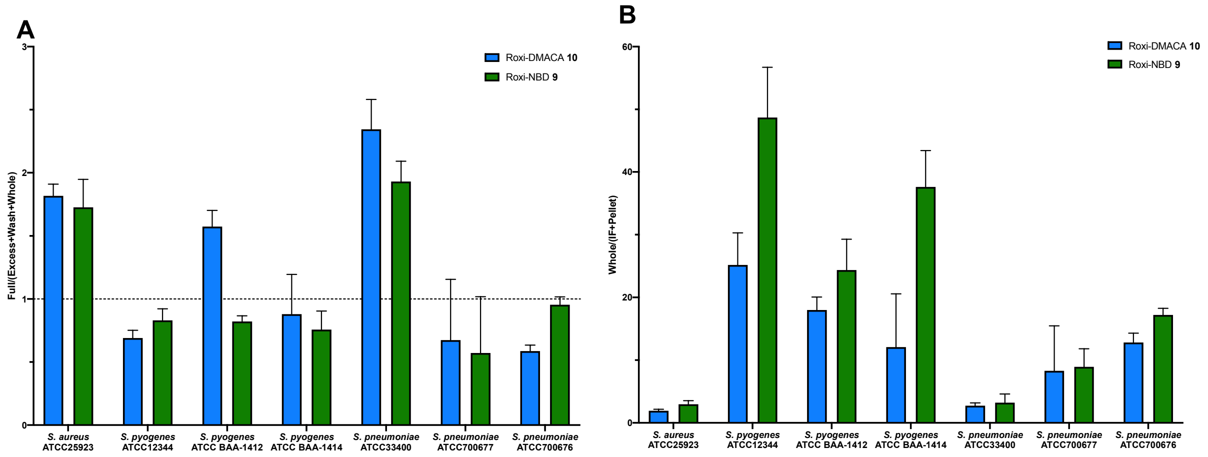

Figure S4: A: Ratio of scaled Full/(Excess + Wash + Whole) fluorescence of roxi-C_4-Tz-DMACA **10** and roxi-C_4-Tz-NBD **9** in different bacteria; **B:** Ratio of scaled Whole/(IF+ Pellet) fluorescence of roxi-C_4-Tz-DMACA **10** and roxi-C_4-Tz-NBD **9** in different bacteria

It is posited that this is due to occlusion of signal due to the high turbidity of the sample at this stage suppressing the true fluorescence. When calculating the total fluorescence of the incubation, the sum of the excess, wash, and whole aliquots has therefore been used, which is nearly identical to the full aliquot in bacteria unaffected by this phenomenon.

A related trend was observed when comparing the whole aliquot to the sum of the IF and pellet aliquots in some species. In the majority of cases (ATCC 33400, BAA-1414, 700676), the sum of the lysis fractions' fluorescence was very similar to the whole aliquot (**Figure S4, B**). Deviations from this were, however, observed, with the whole signal being suppressed in ATCC 25923 (*S. aureus*) and boosted in ATCC 12344 and BAA-1412. This boosting occurs in both susceptible and resistant *S. pyogenes*, indicating that this may be a species-specific phenomenon. Given this inconsistency, when calculating the proportion of probe present in the

different lysis fractions, the sum of the IF and pellet aliquots has been taken as a measure of the whole.

The complete processed results of the assay are shown in **Figure S5**.

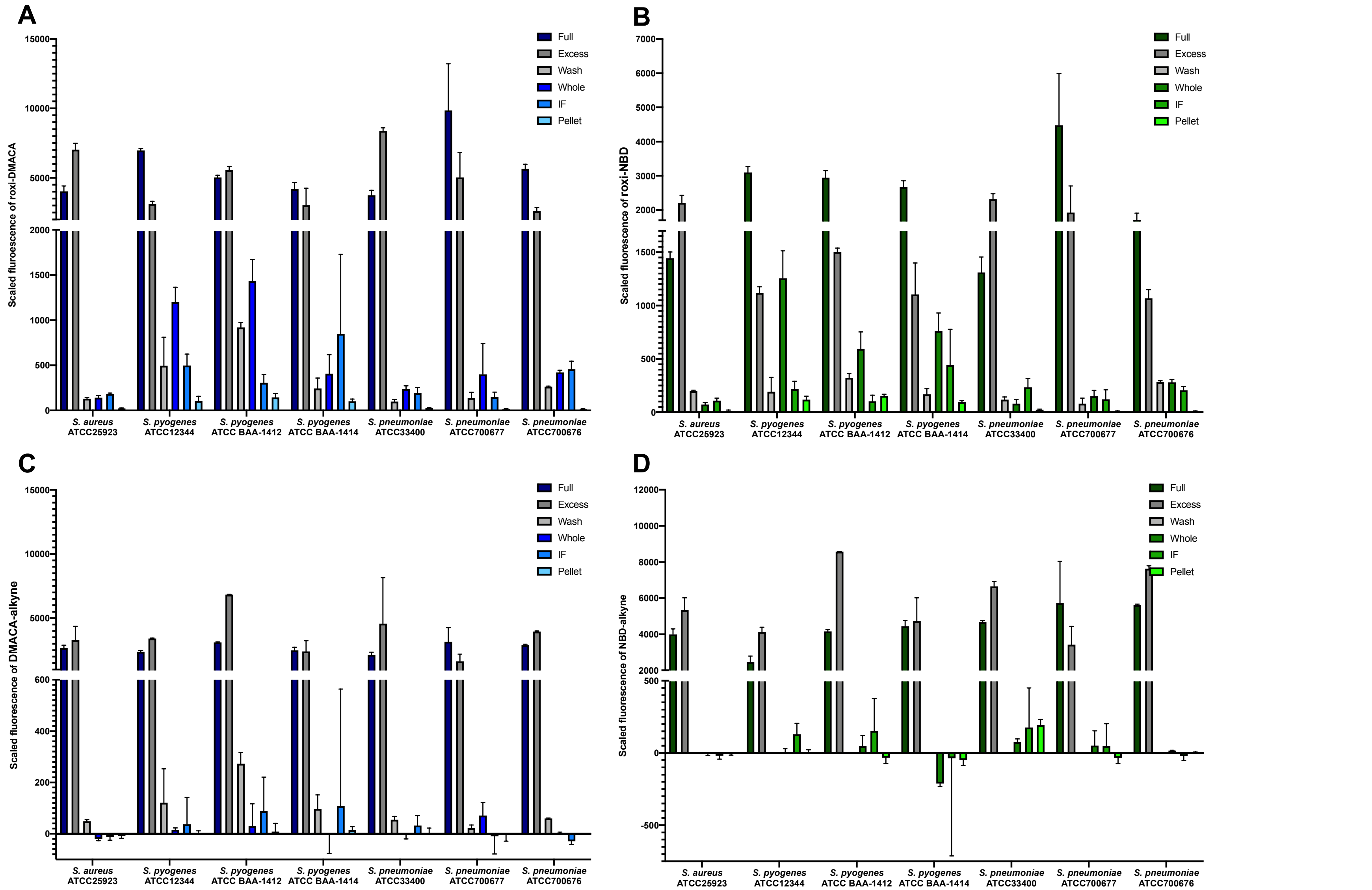

Figure S5: Scaled fluorescence of **A:** roxi-C_4-Tz-DMACA **10**; **B:** roxi-C_4-Tz-NBD **9; C:** DMACA-alkyne **8**; and **D:** NBD-alkyne **7** in aliquots (full, excess, wash, whole, IF, pellet) of different bacteria: susceptible *S. aureus* and susceptible and resistant *S. pyogenes* and *S. pneumoniae*

As mentioned in the main discussion, the two fluorescent macrolides showed similar uptake to the whole, but further, the roxi-C_4-Tz-NBD **9** tending to have a greater uptake. In *S. pyogenes*, resistant bacteria exhibited a roughly halved uptake to the whole as compared to susceptible (**Table S6**). In contrast, ATCC 700676 resistant *S. pneumoniae* (*mef(e)*+ exhibited significantly increased uptake of both probes (**10**, $p = 0.0004$; **9**, $p = 0.0002$). It is unclear why resistant bacteria would take up the probes to a greater degree than their susceptible counter-part, though non-efflux resistance mechanisms may be predominating. Statistical significance was determined by t-test, using the Holm-Sidak method, with an alpha of 0.05.

Table S6: Percentage of fluorescence in the whole aliquot from reconstructed full

			**Average Whole/(Excess+Wash+Whole)**			
**Species**	**ATCC**	**S/R***	**10**	**9**	**8**	**7**
S. aureus	25923	S	2%	3%	<1%	<1%
S. pyogenes	12344	S	25%	49%	<1%	1.1%
	BAA-1412	R	18%	24%	<1%	<1%
	BAA-1414	R	12%	38%	<1%	<1%
S. pneumoniae	33400	S	3%	3%	<1%	<1%
	700677[a]	*R*	*8%*	*6%*	*5%*	*1.8%*
	700676[b]	R	13%	17%	<1%	<1%

*S: susceptible, R: resistant; [a]erm(B)+; [b]mef(e)+; italics: highly variable due to poor absorption

References

[1] J. C. Gasc, S. Gouin D'Ambrieres, A. Lutz, J. F. Chantot, *J. Antibiot.* **1991**, *44*, 313-330.

[2] W. Phetsang, M. A. T. Blaskovich, M. S. Butler, J. X. Huang, J. Zuegg, S. K. Mamidyala, S. Ramu, A. M. Kavanagh, M. A. Cooper, *Bioorg. Med. Chem.* **2014**, *22*, 4490-4498.

[3] S. A. Cochrane, X. Li, S. He, M. Yu, M. Wu, J. C. Vederas, *J. Med. Chem.* **2015**, *58*, 9779-9785.

[4] P. Wang, L. Robert, J. Pelletier, W. L. Dang, F. Taddei, A. Wright, S. Jun, *Current Biol.* **2010**, *20*, 1099-1103.

[5] S. Pagliara, C. Chimerel, R. Langford, D. G. A. L. Aarts, U. F. Keyser, *Lab on a Chip* **2011**, *11*, 3365-3368.

[6] H. Okusu, D. Ma, H. Nikaido, *J. Bacteriol.* **1996**, *178*, 306.
